# Neuronal Cell Fate Specification by the Convergence of Different Spatiotemporal Cues on a Common Terminal Selector Cascade

**DOI:** 10.1371/journal.pbio.1002450

**Published:** 2016-05-05

**Authors:** Hugo Gabilondo, Johannes Stratmann, Irene Rubio-Ferrera, Irene Millán-Crespo, Patricia Contero-García, Shahrzad Bahrampour, Stefan Thor, Jonathan Benito-Sipos

**Affiliations:** 1 Departamento de Biología, Universidad Autónoma de Madrid, Cantoblanco, Madrid, Spain; 2 Department of Clinical and Experimental Medicine, Linköping University, Linköping, Sweden; University of Cambridge, UNITED KINGDOM

## Abstract

Specification of the myriad of unique neuronal subtypes found in the nervous system depends upon spatiotemporal cues and terminal selector gene cascades, often acting in sequential combinatorial codes to determine final cell fate. However, a specific neuronal cell subtype can often be generated in different parts of the nervous system and at different stages, indicating that different spatiotemporal cues can converge on the same terminal selectors to thereby generate a similar cell fate. However, the regulatory mechanisms underlying such convergence are poorly understood. The Nplp1 neuropeptide neurons in the *Drosophila* ventral nerve cord can be subdivided into the thoracic-ventral Tv1 neurons and the dorsal-medial dAp neurons. The activation of Nplp1 in Tv1 and dAp neurons depends upon the same terminal selector cascade: *col*>*ap*/*eya*>*dimm*>*Nplp1*. However, Tv1 and dAp neurons are generated by different neural progenitors (neuroblasts) with different spatiotemporal appearance. Here, we find that the same terminal selector cascade is triggered by *Kr*/*pdm*>*grn* in dAp neurons, but by *Antp*/*hth*/*exd*/*lbe*/*cas* in Tv1 neurons. Hence, two different spatiotemporal combinations can funnel into a common downstream terminal selector cascade to determine a highly related cell fate.

## Abstract 2

A study of neuropeptide neurons in the *Drosophila* nervous system reveals that two different combinations of spatiotemporal cues—active in different progenitors—converge on a common terminal selector gene to trigger a similar neuronal subtype identity.

## Author Summary

A fundamental challenge in developmental neurobiology is to understand how the great diversity of neuronal subtypes is generated during nervous system development. Neuronal subtype cell fate is established in a stepwise manner, starting with spatial and temporal cues that confer distinct identities to neural progenitors and trigger expression of terminal selector genes in the early-born neurons. Terminal selectors are those that determine the final neuronal subtype cell fate. Intriguingly, similar neuronal subtypes can be generated by different progenitors and under the control of different spatiotemporal cues; thus, we wondered how such convergence is achieved. To address this issue, we have decoded the specification of two highly related neuropeptide neurons, which are generated at different locations and time-points in the *Drosophila* nervous system. We find that two different combinations of spatiotemporal cues, in two different neural progenitors, funnel onto the same terminal selector gene, which in turn activates a shared regulatory cascade, ultimately resulting in the specification of a similar neuronal cell subtype identity.

## Introduction

During nervous system development, vast numbers of different neuronal subtypes are generated, and understanding the process of cell fate specification remains a major challenge. Studies have shown that establishment of distinct neuronal identities requires complex cascades of regulatory information, starting from spatial and temporal selector genes [1] and feeding onward to terminal selector genes [2,3], often acting in combinatorial codes to dictate final and unique cell fate [4–6]. One particularly intriguing regulatory challenge pertains to the generation of highly related neuronal subtypes in different regions of the central nervous system (CNS). Examples are plentiful and include e.g., various groups of dopaminergic and serotonergic neurons in the mammalian CNS [7,8], as well as neuropeptide-producing neurons in many systems [9,10]. The appearance of highly related neurons in different regions and at distinct developmental time-points clearly indicates that different spatial and temporal cues can converge to trigger the same terminal selector code, to thereby trigger a similar final cell fate. However, the underlying mechanisms are unclear.

**Fig 1:**
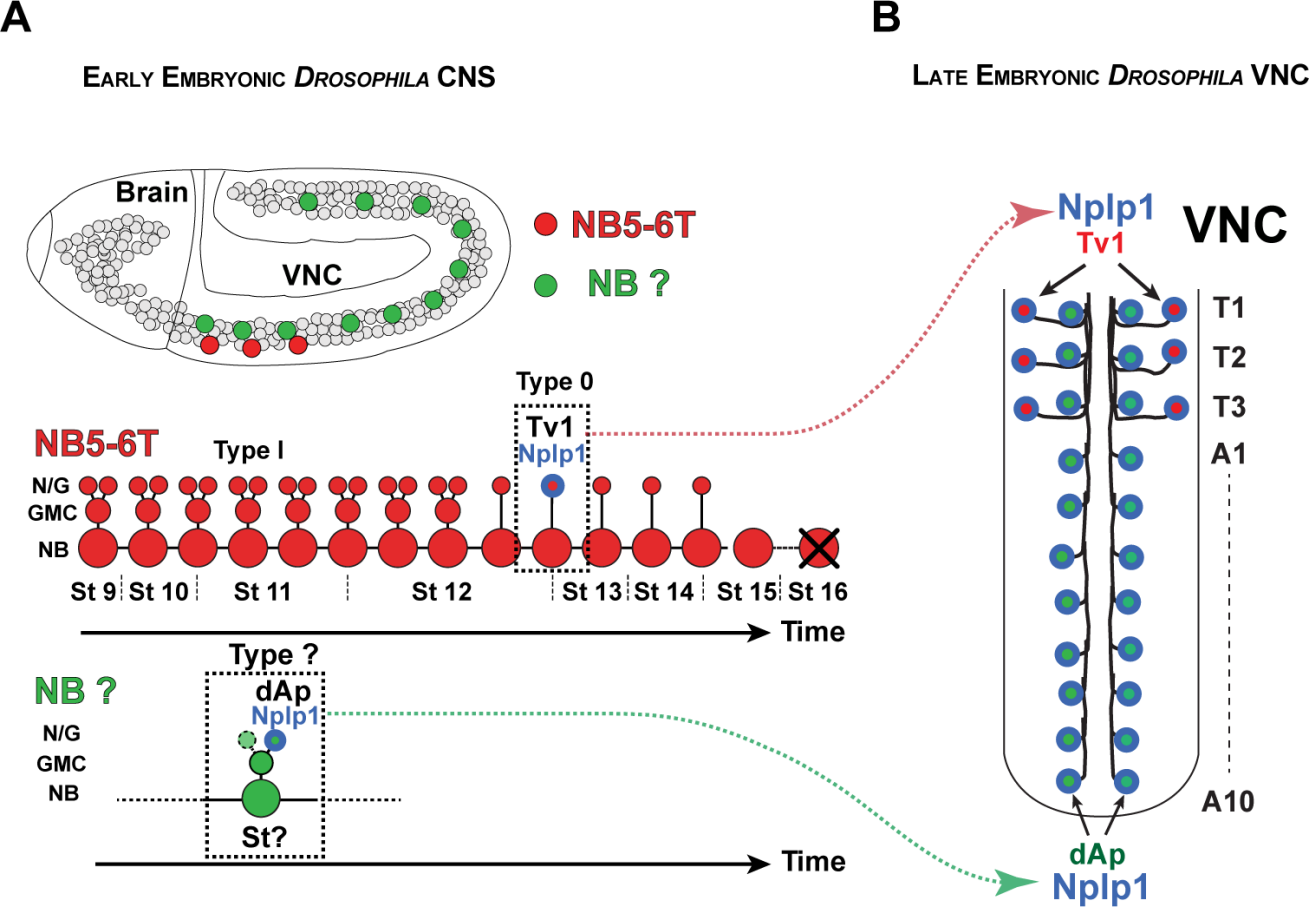
dAp and Tv1 neurons in the *Drosophila* VNC. (A) Lateral view of early embryonic *Drosophila* CNS, showing NB5-6T in the three thoracic segments (red) and the NB that gives rise to the dAp cells (green). Model of NB5-6T lineage, with the Tv1 neuron (red/blue). (B) Model of late embryonic *Drosophila* VNC (air-filled trachea [AFT] stage), depicting the Ap clusters in the thoracic segments and the dAp cells located in the segments T1-A10.

In the developing *Drosophila* ventral nerve cord (VNC), two distinct sets of neurons selectively express the neuropeptide Nplp1: dAp and Tv1. Both subtypes express the LIM-homeodomain transcription factor Apterous (Ap; mammalian Lhx2a/b) and the transcription co-factor Eyes absent (Eya; mammalian Eya1-4). dAp neurons constitute a dorsal-medial set of bilateral neurons running the length of the ventral nerve cord, while Tv1 neurons are located ventrolaterally in the three thoracic segments (Fig 1A and 1B). Both dAp and Tv1 project axons ipsilaterally and anteriorly, and join a common Ap fascicle [11,12]. While it is possible that other aspects of their cell fate are different, their common neuropeptide expression and axonal projections suggest that dAp and Tv1 can be grouped into a highly related, if not identical, neuronal subtype. A number of regulatory genes and pathways acting in the specification of the Tv1 neurons have been elucidated [6,11,13–20]. These studies reveal that Tv1 cell fate depends upon a feedforward cascade in which spatial cues, provided by Hox and Hox cofactor input (Antp, Exd and Hth), and temporal cues, provided by the temporal factor Cas, activate a *col*→*ap*/*eya*→*dimm* terminal selector cascade. This selector cascade ultimately results in the activation of Nplp1 neuropeptide expression. dAp neurons depend upon the same *col*→*ap*/*eya*→*dimm* terminal selector cascade as Tv1. However, dAp neurons are not restricted to thoracic segments, but rather are distributed throughout the VNC (Fig 1A and 1B). In addition, they are born at an earlier stage than Tv1 [12]. Furthermore, while Tv1 is generated by NB5-6T, the lineage that generates dAp is unknown [6]. Not surprisingly, the upstream spatial and temporal cues that trigger the terminal selector cascade in the Tv1 neuron do not affect the dAp cells [14,17]. Thus, the dAp and Tv1 cells represent a unique scenario for addressing how neurons generated by different neuroblasts (NBs) and with different spatial and temporal regulators can activate the identical terminal selector cascade to ultimately dictate a highly related, if not identical, neuronal subtype identity.

Here, we identify the NB generating the dAp neurons as NB4-3 and find that these dAp neurons are generated during an earlier time in development than Tv1, dictated by the temporal factors Kr and Pdm. Hence, the same terminal selector cascade (*col*→*ap*/*eya*→*dimm*) is triggered by distinct spatial and temporal cues in two different neuroblasts. Additionally, we find two crucial and specific factors refining the action of those spatiotemporal selectors: the GATA factor Grain (Grn), acting in dAp neurons, and the Ladybird early factor (Lbe), in Tv1 neurons. Thus, the *col*→*ap*/*eya*→*dimm* terminal selector cascade is triggered by the Cas/Exd/Hth/Antp/Lbe spatiotemporal code in NB5-6T, but by the Kr/Pdm/Grn code in NB4-3. These results demonstrate that distinct spatiotemporal combinatorial codes can converge onto a common terminal selector cascade. Because the generation of highly related neurons in different regions of the CNS and at distinct time-points represents a common feature of many animal systems, the regulatory logic outlined here is likely to be widespread.

## Results

### dAp is Generated by NB4-3 in an Early Temporal Window

**Fig 2:**
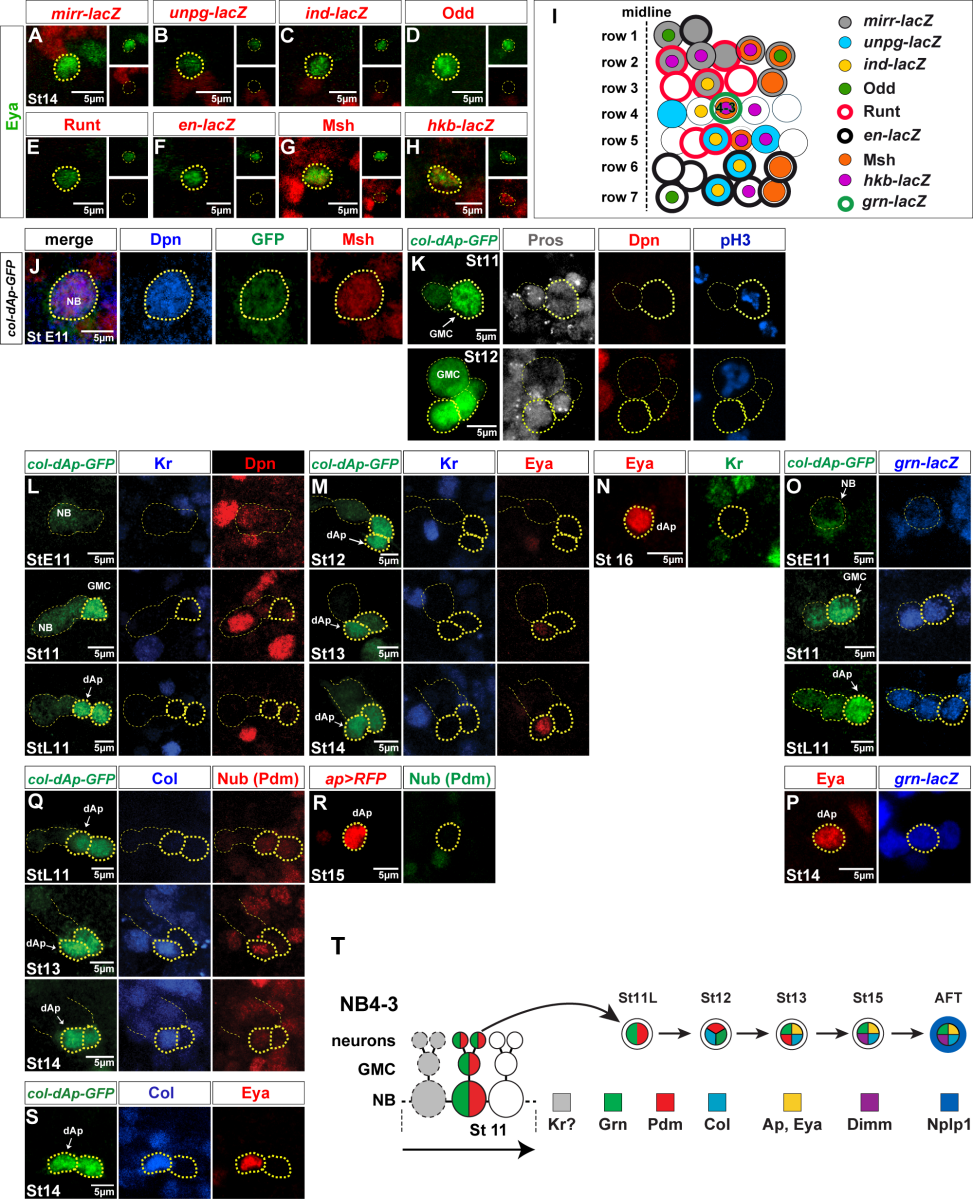
Dorsal Ap cells are generated by NB4-3. (A–H) NB marker expression, either direct antibody stain or βgal stain of lacZ constructs, costained with Eya to identify the progenitor of the dAp cell. (A–F) None of the markers overlap with the Eya expression of the dAp cell. (G) Msh staining overlaps with the Eya expression in the dAp neuron. (H) βgal expression from the *hkb-lacZ* construct shows overlap with the Eya expression in the dAp cell. (I) Model of a hemisegment during mid embryogenesis, showing the NBs with their different markers. Combination of immunostains for specific markers made it possible to rule out certain NBs as progenitors of the dAp cell. The dAp cell is positive for *hkb-lacZ* and Msh, which indicates that NB4-3 is the progenitor of dAp cells. (J) NB staining for Msh shows overlap with Dpn and the col-dAp-GFP enhancer, which indicates that the NB generating the dAp cell, is NB4-3. (K) Expression of GFP, Pros, Dpn, and pH3 at St11 and St12 shows a pH3/Pros positive and Dpn negative cells (St11 thick yellow dashed circle) overlapping with the GFP expression from the *col-dAp-GFP* enhancer construct, which suggests that the dAp cell is born in a type I division. (L) GFP, Kr, and Dpn expression at different stages (yellow dashed circles). At StE11, Dpn and GFP are expressed in the NB, but Kr is not detectable. At St11, the NB 4-3 lineage progresses (based on the GFP signal from the enhancer construct), Dpn is still detectable, but Kr is not expressed. By StL11, two strong GFP positive cells are detectable and GFP expression is evident in a cell in the previous NB location, but without a Dpn signal. (M) GFP, Kr, and Eya expression at St12, St13, and St14 shows that one of the cells expressing strong GFP is the dAp cell and turns on Eya expression by stage 13 (thick yellow dashed circle). (N) Kr is not expressed in the dAp cells at stage 16 (yellow dashed circles). (O) Costaining for GFP and βgal of the *col-dAp-GFP* enhancer together with *grn-lacZ*, shows that *grain*, one of the critical factors for the dAp specification is expressed in the NB4-3 lineage (yellow dashed circles). (P) Staining for Eya and βgal shows that *grn* is expressed in the dAp cells by stage 14 (yellow dashed circles). (Q) GFP (*col-dAp-GFP*), Col, and (Nub) Pdm expression at different stages in the NB4-3 lineage reveals that GFP is detectable prior to endogenous Col expression at StL11. At this stage, Nub (Pdm) starts being expressed in the two strong GFP positive cells (thick yellow dashed circles). At StM13 the GFP signal remains strongly expressed; furthermore, Col and Nub (Pdm) are expressed robustly. Of note, Col and Nub (Pdm) expression overlaps in one of the strong GFP positive cells (thick yellow dashed circles). At St14, Col expression is still strong, whereas Nub (Pdm) expression is downregulated (thick yellow dashed circles); Col, GFP, Nub (Pdm) expressing cell is the dAp cell. (R) Nub (Pdm) is not expressed in the dAp cells at stage 15 (yellow dashed circles). (S) GFP (*col-dAp-GFP*), Col, and Eya show an overlap in the dAp cell at St14. (T) Part of the lineage model of the NB 4–3. At StE11, Kr was not expressed in the NB (L). Still, we find Grn and Nub expression in the NB and the daughter cells. Together with the positive pH3 staining, prior to birth of the dAp cell, this model suggests that the dAp cell is born in a type I division mode by St12 and subsequently activates Col, Eya, and by later stages Dimm and Nplp1. Genotypes: (A) *mirr-lacZ*. (B) *unpg-lacZ*. (C) *ind-lacZ*. (D, E) *OregonR*. (F) *en-lacZ*. (G) *OregonR*. (H) *hkb-lacZ*/+. (J) *col-dAp-GFP*; *col-dAp-GFP*. (L, M, Q, and S) *col-dAp-GFP*; *col-dAp-GFP*. (N) *OregonR*. (O and P) *col-dAp-GFP*/+; *col-dAp-GFP*/*grn-lacZ*. (R) *ap-Gal4*, UAS-*mRFP*/CyO.

Previous work demonstrated that the terminal selector cascade composed by *col*→*ap*/*eya*→*dimm* is critical for the Nplp1 terminal cell fate both in dAp and Tv1 neurons, and can trigger this fate broadly in the CNS when combinatorially misexpressed [6,11,13–20]. Tv1 neurons have been most extensively studied, and their NB origin is well understood [14,17]. They are generated at the end of the NB5-6T lineage, under a Castor (Cas) temporal window and through a type 0 division mode (Fig 1A) [14,21]. dAp neurons arise from a distinct, previously unknown NB lineage. Thus, we began by identifying the progenitor NB that gives rise to dAp neurons, utilizing sets of markers that identify most, if not all, of the 30 NBs generated in each hemisegment [22–26]. Eya expression commences in dAp at St13, and using Eya together with a number of NB markers, we found that dAp neurons are generated by NB4-3 (Fig 2A–2I). To follow the development of this lineage, we made use of a *col* enhancer that drives reporter expression selectively in the dAp neuron, as well as in the NB4-3 and parts of the lineage (Fig 2J and 2K). NB4-3 is known to delaminate at St late 11 [25], and we can observe the lineage using *col-GFP* from this stage and onward. We mapped the expression of the temporal genes, and as anticipated from the early birth of dAp, evident by Eya expression, we did not find expression of the late temporal factor Cas (S3A Fig). We did not observe expression of the early temporal factor Kr (Fig 2L–2N). Because both *hb* and *Kr* mutants affect dAp specification (see below), we envision that Hb and Kr are expressed in the NB4-3 prior to the onset of *col-GFP*. One of the two “middle” temporal factors Pdm1 (Nubbin [Nub], which together with Pdm2 we collectively refer to as Pdm1/2) was, however, expressed in several cells in the NB4-3 lineage (*col-GFP* cells) (Fig 2Q). When Col and Eya are turned on, we can identify Nub expression specifically in the early dAp neuron itself at St13, to subsequently be downregulated at St15 (Fig 2Q and 2R). Using anti-phospho-Ser10 on Histone 3 (pH3), we were able to monitor cell divisions in the NB4-3 lineage, which revealed that dAp is born by a ganglion mother cell (GMC) and is hence generated in a type I proliferation window (Figs 2K and S3B). In order to unambiguously show that dAp comes from a type I lineage, we analyzed the dAp neuron in a *sanpodo* (*spdo*) mutant background. Corroborating the notion deduced with the pH3 analysis, we observe two dAp neurons in that mutant background (S3B Fig). Therefore, dAp is born in a type I proliferation window.

In a screen for regulators and specific markers of dAp cell fate, we identified the GATA factor Grain (Grn) as being expressed in the dAp cell (see below), and could hence use a *grn-lacZ* reporter to map the NB4-3 lineage. We found that *grn-lacZ* expression is concomitant with Pdm and hence precedes Col, being turned on in the GMC that generates the dAp cell (Fig 2O). We further observed *grn-lacZ* expression in dAp neurons at all later embryonic stages (Fig 2P).

In summary, we map the origin of dAp to NB4-3 and find that it is born in the middle of this lineage. At the stage when NB4-3 generates the GMC that in turn will divide to generate the dAp neuron, it expresses Pdm and Grn (Fig 2O and 2Q). Hence, dAp and Tv1 are lineage-unrelated neurons, generated in different temporal windows, mid versus late, and during two different proliferation modes, type I versus type 0.

### The Early Born dAp Neurons Depend upon Early Temporal Genes

**Fig 3:**
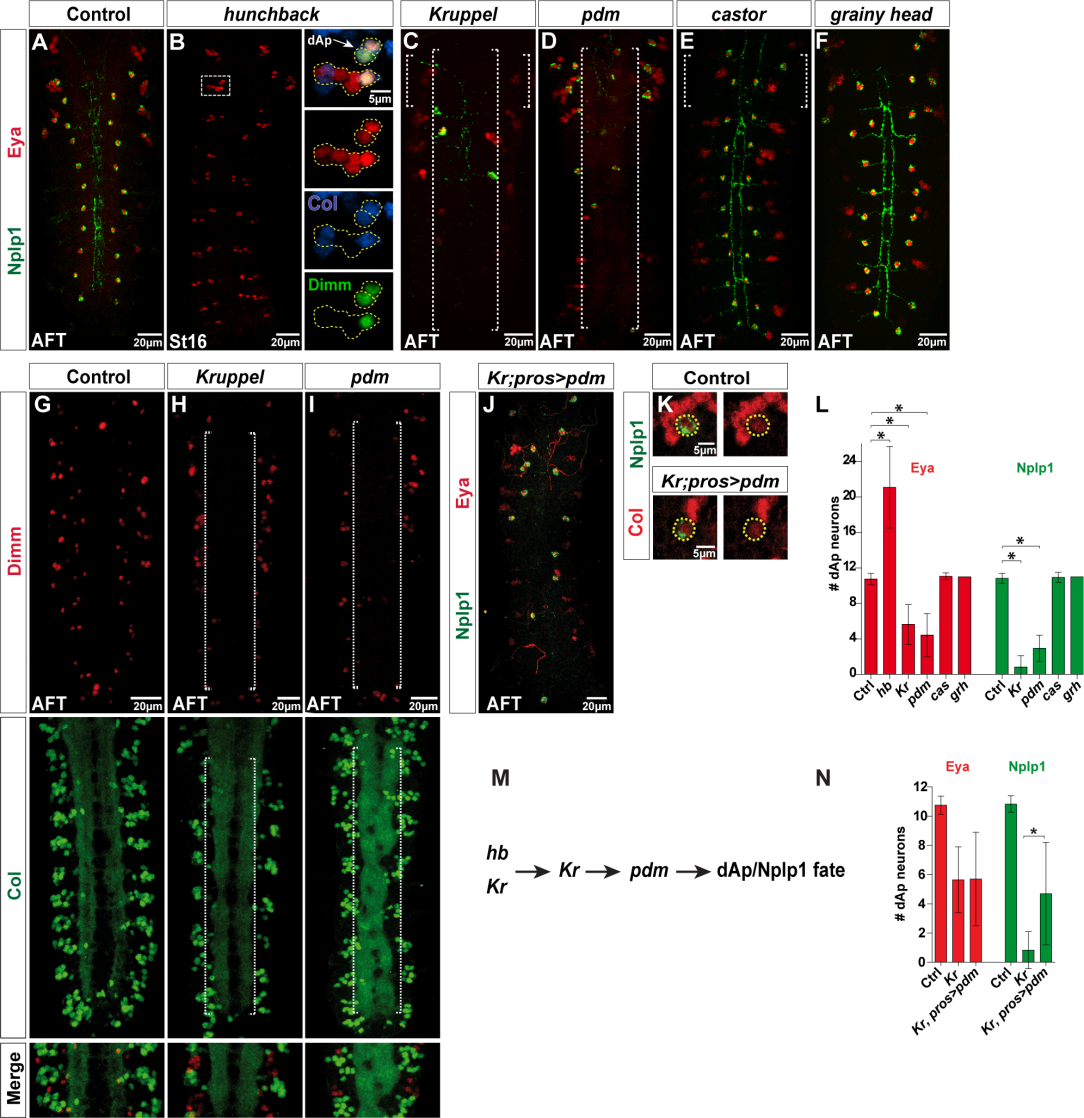
Early temporal genes are critical for dAp specification. (A–F) Expression of Eya and Nplp1 in control and temporal mutants, at St16 or AFT. (B) In *hb* mutants (boxed area), we observe two dorsal Ap cells (yellow dotted circles), which both express Eya, Col, and Dimm. Quantification of Nplp1 positive dAp cells in *hb* mutants fails since *hb* mutants do not develop into stage AFT at which Nplp1 is expressed. (C and D) Both *Kr* and *pdm* mutants show decreased numbers of Eya and Nplp1 expressing dAp cells (long dotted brackets). (E and F) Eya and Nplp1 expression in *cas* and *grh* mutants is not affected. (G–I) Dimm and Col expression shows a loss of both factors in the dAp cells in *Kr* and *pdm* mutants (long dotted brackets). (J) Cross-rescue of *Kr* mutants by *UAS-pdm* from *pros-Gal4* does not rescue Eya expression in dAp cells, but can partially rescue Nplp1 expression. (K) Col and Nplp1 expression in dAp cells of control and *Kr* mutants expressing *pdm* from *pros-Gal4* shows that *pdm* can restore the Col and Nplp1 expression in *Kr* mutants. (L) Quantification of Eya and Nplp1 positive dAp neurons in temporal mutants (n > 10; asterisk denotes *p* < 0.05; Student´s two-tailed *t* test; see S1 Data). (M) Genetic model of the dAp specification cascade, showing that the early temporal genes *Kr* and *pdm* act to specify the dorsal Ap cell fate. (N) Quantification of Eya and Nplp1 positive dAp neurons in (n > 10 VNC; asterisk denotes *p* < 0.05; Student´s two-tailed *t* test; see S1 Data). Numbers of Nplp1 positive dAp neurons in *Kr* mutants expressing *pdm* are significantly increased compared to *Kr* mutants. Genotypes: (A) *OregonR*. (B) *hb*^*P1*^, *hb*^*FB*^. (C, H) *Kr*^*1*^, *Kr*^*CD*^. (D, I) *Df(2L)ED773*. (E) *cas*^*Δ1*^*/ cas*^*Δ1*^. (F) *grh*^*IM*^*/grh*^*IM*^. (G) *OregonR*. (J, and K) *Kr*^*1*^, *Kr*^*CD*^; *pros-Gal4/UAS-nub*.

Having identified that dAp is born early in NB4-3, we next tested the expression of Nplp1/Eya markers in mutants for the temporal genes. In the early temporal mutant *hb*, we observed an apparent duplication of dAp neurons, evident by Col, Eya, and Dimm expression (Fig 3B and S2). In *Kr* mutants we found a reduction of Col, Eya, Dimm, and Nplp1 expressing cells (Fig 3C, 3H and 3L). As anticipated from the expression of Pdm in the GMC generating the dAp cells, and in the dAp cells themselves, we also observed loss of dAp neuron markers in *pdm* mutants (*Df(2L)ED773*, a genomic deletion that removes both *nub* and *Pdm2*) (Fig 3D, 3I and 3L). Previous studies revealed that *Kr* regulates Pdm [27,28]; to address their relationship with regards to dAp specification, we attempted to cross-rescue *Kr* mutants with *UAS-pdm*, driven from the NB driver *pros-Gal4*. This experiment revealed a partial rescue, evident by expression of Col and Nplp1, while Eya was not significantly rescued (Fig 3J, 3K and 3N and S1 Data). As anticipated from previous studies [14], the late temporal gene *cas* specifically affected Tv1 and not dAp, while *grh* did not affect either cell (Fig 3E, 3F and 3L and S1 Data). To determine if dAp neurons undergo cell death in *Kr* and *pdm* mutants, we combined these mutants with the cell death mutant *Df(3R)H99*, which removes all embryonic cell death [29]. We did not, however, note any rescue of dAp cell in these double mutants (S7A and S7B Fig). We conclude that dAp neurons, which are born in an early temporal window, depend upon the early temporal genes *hb*, *Kr*, and *pdm* for their specification. In contrast, Tv1 neurons, which are born late, depend upon the late temporal gene *cas*.

### *grain* is Necessary for dAp but not for Tv1 Specification

**Fig 4:**
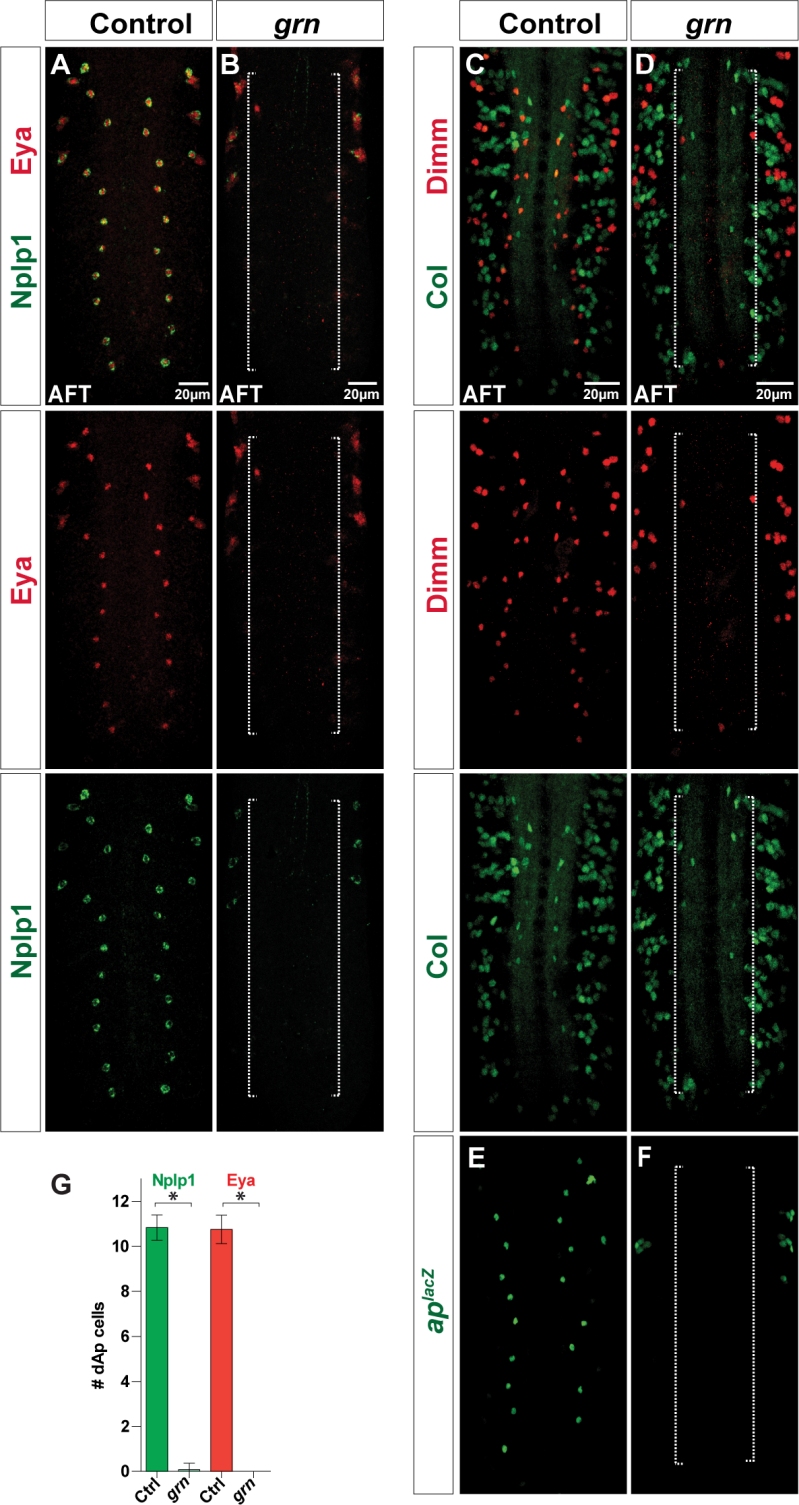
*grain* is critical for dAp specification. (A, B) Eya and Nplp1 expression in VNCs at stage AFT. In *grn* mutants, Eya and Nplp1 expression in dAp cells is almost completely lost (long dotted bracket). (C-F) Expression of Dimm, Col and βgal (*ap*^*rK568*^) in control and *grn* mutants. In *grn* mutants all three markers are strongly downregulated, specifically in dAp cells (long dotted brackets). In contrast, expression in Tv1 cells is unperturbed. (G) Quantification of Nplp1 and Eya expressing dAp cells in control and *grn* mutant VNCs (n > 10 VNCs; asterisks denote significant difference in *grn* mutants compared to control; *p* < 0.05, Student´s two-tailed *t* test; see S1 Data). Genotypes: (A) *OregonR*. (B) *grn*^*7L12*^*/grn*^*SPJ9*^. (C, E) *ap*^*rK568*^*/+*. (D, F) *ap*^*rK568*^*/+; grn*^*SPJ9*^*/grn*^*7L12*^.

The distinct NB origin and spatiotemporal generation of dAp and Tv1 demonstrates that two different sets of spatiotemporal inputs can converge upon the same terminal selector cascade (*col*→*ap*/*eya*→*dimm*), which triggers Nplp1 expression. Although the temporal factors are selectively expressed at different points of the lineage development, they are broadly spatially expressed in most NBs during neurogenesis [27,28]. Hence, we predicted the existence of additional upstream spatially-defining regulatory genes acting with the *Kr* and *pdm* temporal genes. In order to identify such additional upstream cues, we analysed a number of mutants for changes in Nplp1 expression in dAp but not in Tv1 cells or vice-versa (see Materials and Methods). In the case of the dAp neurons, one mutant identified in this survey was *grain* (*grn*), which encodes a GATA transcription factor known to be dynamically expressed in the developing VNC [30]. Our expression mapping of NB4-3 revealed expression of *grn*^*lacZ*^ in the NB at StE11, in the GMC at StL11, and in early dAp cells from St14 and onward to St16 (Figs 2O, 2P and S4A). Addressing the function of *grn*, we found that several *grn* allelic combinations all displayed complete loss of Col, Eya, *ap*^*lacZ*^, Dimm, and Nplp1 expression in dAp, but not in Tv1 neurons (Fig 4A–4G and S1 Data). To determine if dAp neurons undergo cell death in *grn* mutants, we expressed the cell death blocker p35. This did not, however, result in rescue of dAp cells (S7C Fig). Thus, the *grn* mutant analysis indicates that *grn* is an early factor, acting upstream of *col*, in the dAp specification cascade. Strikingly, *grn* is not involved in triggering this cascade in the Tv1 neuron (Figs 4A–4D, S4A and S4B).

### *grain* Acts at an Early Stage of dAp Specification

**Fig 5:**
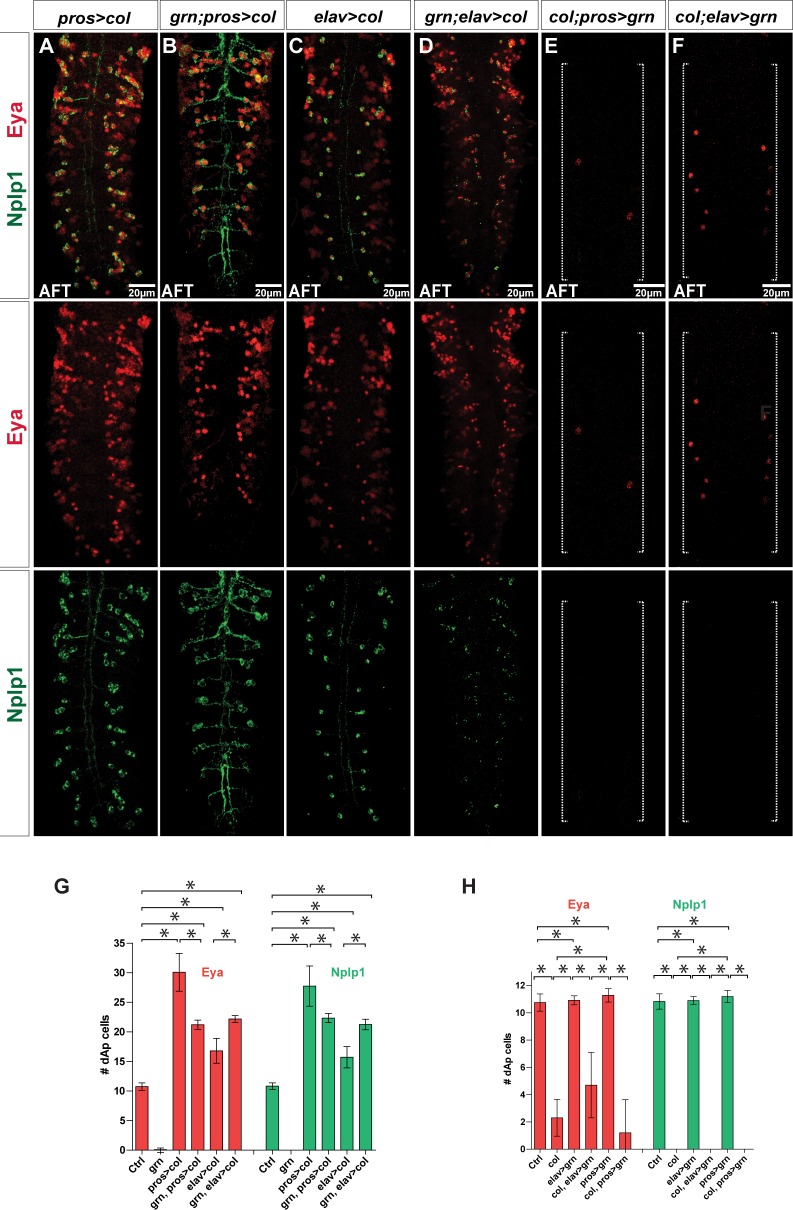
Cross-rescue reveals that the primary role of *grn* is to activate *col*. (A-D) Eya and Nplp1 expression in *grn* mutants cross-rescued with *UAS-col*, from either *pros-Gal4* or *elav-Gal4*. Both early and late misexpression of *col* rescues the *grn* mutant phenotype and results in ectopic expression of Eya and Nplp1 positive dAp cells. (E and F) In contrast, cross-rescue of *col* mutants with *UAS-grn*, from either *pros-Gal4* or *elav-Gal4*, fails to rescue the *col* mutant phenotype (long dotted bracket). (G) Quantification of Eya and Nplp1 positive dAp cells shows a significant increase when *col* is misexpressed in *grn* mutants from either *pros-Gal4* or *elav-Gal4* compared to control VNCs (n = 8 VNCs for *elav>col* and *grn; elav>col* for all others, n > 10 VNCs; asterisks denote *p* < 0.05, Student´s two-tailed *t* test). (H) Quantification of Eya and Nplp1 positive dAp cells in *col* mutants with *grn* misexpression shows that *grn* does not rescue the *col* mutant phenotype (n = 7 VNCs for *pros>grn*, Eya cell quantification; for all others, n > 10 VNCs; asterisks denote *p* < 0.05, Student´s two-tailed *t* test; see S1 Data). Genotypes: (A) *UAS-col/pros-Gal4*. (B) *UAS-col/pros-Gal4; grn*^*SPJ9*^*/grn*^*7L12*^. (C) *elav-Gal4/UAS-col*. (D) *elav-Gal4/UAS-col; grn*^*SPJ9*^*/grn*^*SPJ9*^. (E) *col*^*1*^*/col*^*1*^, *UAS-grn; pros-Gal4*/+. (F) *col*^*1*^*/col*^*1*^, *UAS-grn; elav-Gal4*/+.

*col* is a critical determinant of early dAp neuron identity [6], and we find that *grn* acts upstream of *col*. Thus, we next addressed whether all of the *grn* functions in the dAp specification are mediated by *col*. To this end, we re-expressed *col* in *grn* mutants from *Gal4* drivers with different temporal onset: *pros-Gal4* at St10 and *elav-Gal4* at St12 [17,21]. We found robust re-appearance of dAp neurons, showing both the Eya and Nplp1 markers, when we used either the *pros-Gal4* or *elav-Gal4* drivers (Fig 5B, 5D and 5G). As anticipated from previous studies [6], expression of *UAS-col* from either *pros-Gal4* or *elav-Gal4* also triggered a number of ectopic Eya/Nplp1 cells (Fig 5A and 5C). In a reciprocal experiment we tried to cross-rescue dAp cell fate in *col* mutants by expressing *grn* from *elav-Gal4* or *pros-Gal4*. We did not, however, find any rescue of dAp cell specification in these cross-rescues (Fig 5E, 5F and 5H; S5 Fig and S1 Data).

Together, these results suggest that the main, if not the only, role of *grn* in dAp cells is to trigger the expression of *col*, setting in motion the cascade of regulatory events that culminate with the dAp specification.

### *grain* Acts Downstream of the *Kr* and *pdm* Temporal Genes

**Fig 6:**
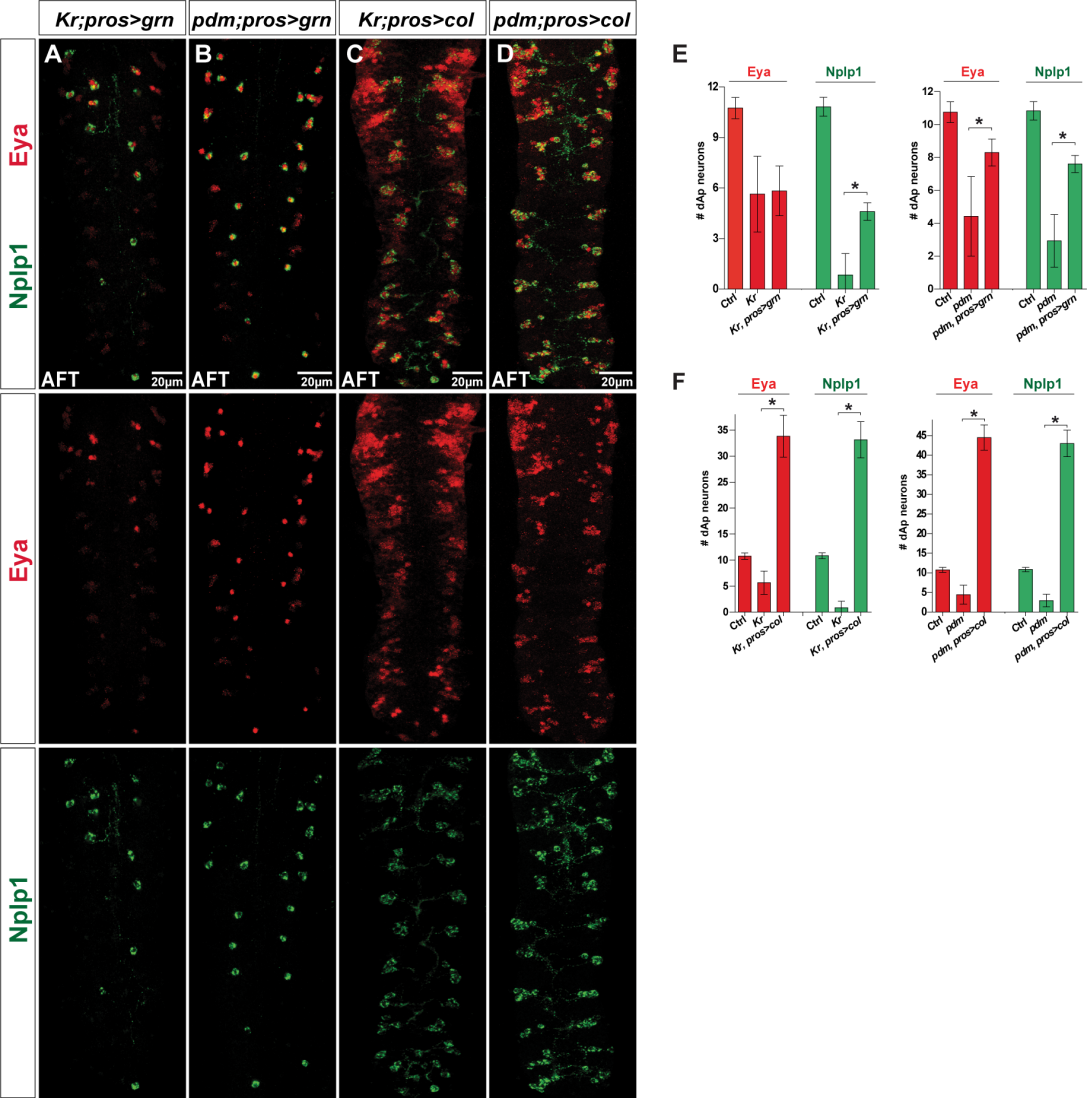
Cross-rescue reveals that *Kr* and *pdm* act upstream of *grn* and *col*. (A–D) Eya and Nplp1 expression in cross-rescue of *Kr* and *pdm* mutants with either *UAS-col* or *UAS-grn* misexpressed from *pros-Gal4*, at stage AFT. (A) While the number of Eya expressing dAp neurons in *Kr* mutants is not restored by misexpression of *grn*, (B) in *pdm* mutants misexpression of *grn* results in partial rescue in numbers of dAp neurons expressing both Eya and Nplp1. (C) Cross-rescue of *Kr* with *col* can fully rescue the mutant phenotype and results in ectopic numbers of dAp neurons expressing both Eya or Nplp1. (D) Cross-rescue of *pdm* with *col* can fully rescue the *pdm* mutant phenotype with respect to Eya and Nplp1 positive dAp neurons, and results in ectopic Eya and Nplp1 expression. (E, F) Quantification of Eya and Nplp1 positive dAp neurons from the different rescue experiments (n = 9 VNCs for *Kr; pros>grn*, n = 8 VNCs for *pdm; pros>col*. For all others n >10 VNCs; asterisk denotes *p* < 0.05, Student´s two-tailed *t* test; see S1 Data). Genotypes: (A) *Kr*^*1*^, *Kr*^*CD*^*; pros-Gal4*/ *UAS-grn*. (B) *Df(2L)ED773; pros-Gal4*/*UAS-grn*. (C) *Kr*^*1*^, *Kr*^*CD*^*; pros-Gal4*/*UAS-col*. (D) *Df(2L)ED773; pros-Gal4*/*UAS-col*.

Our lineage and expression analyses indicated that *grn* acts downstream of the *Kr* and *pdm* temporal genes, and that its primary role is to trigger *col* expression. To further test this notion, we drove the expression of *grn* in *Kr* and *pdm* mutants. In both cases, we found partial rescue of the dAp neurons (Fig 6A, 6B and 6E). Next, we expressed *col* in *Kr* and *pdm* mutants and observed rescue of dAp neurons in both experiments (Fig 6C, 6D and 6F and S1 Data). Misexpression of *UAS-col* again triggered a number of ectopic Eya/Nplp1 cells (Fig 6C and 6D).

These findings indicate that dAp cell fate is specified by a *Kr/pdm>grn>col* cascade, in which the function of *Kr/pdm* is to activate *grn*, and the function of *grn* is to activate *col*. However, the partial rescue of *Kr* by *grn* suggests that *Kr* may be involved in a feedforward manner to regulate *col*.

### *ladybird early* Delimits the Broad Action of the Spatio-temporal Cues Responsible for the Tv1 Specification

**Fig 7:**
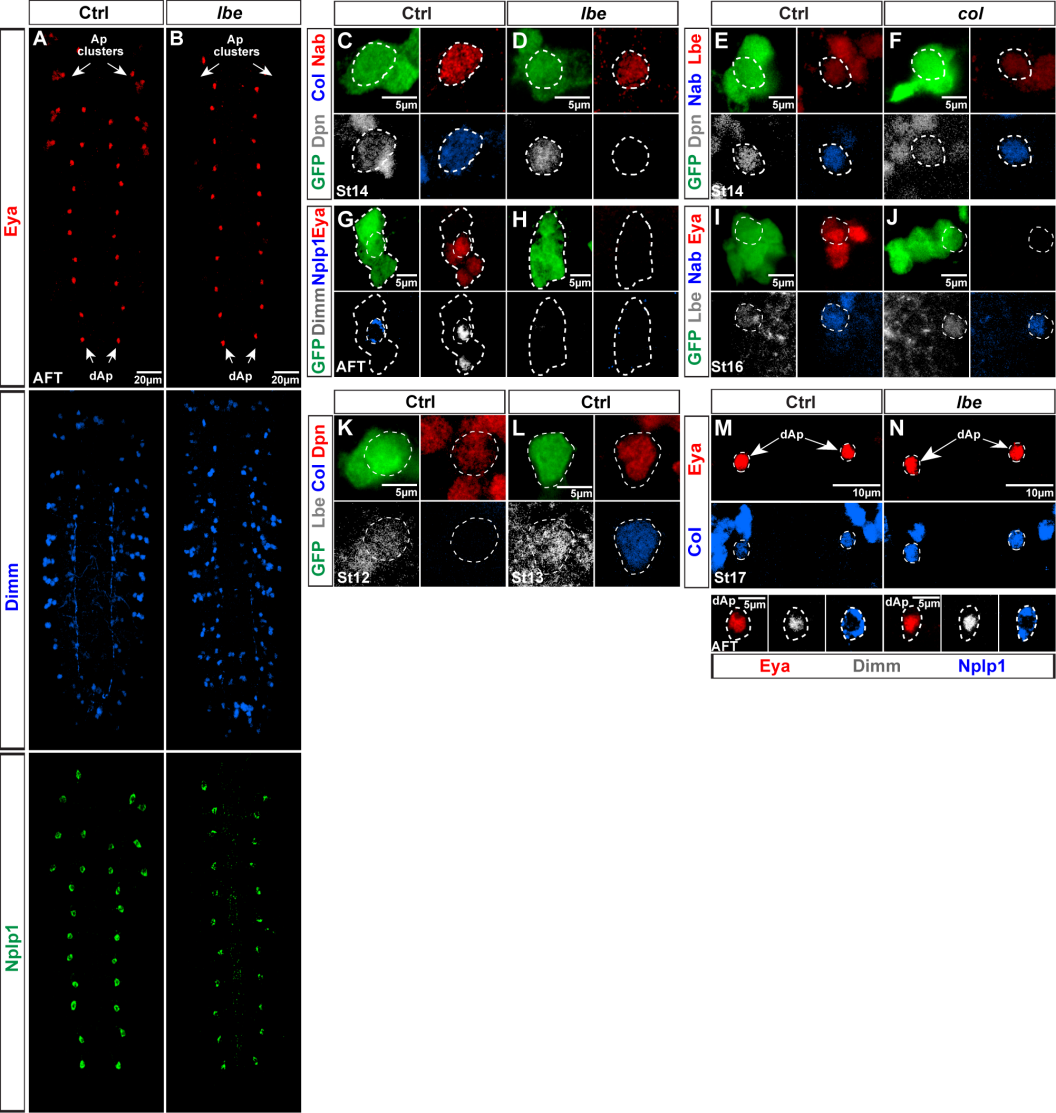
*lbe* is critical for Ap cluster formation and Tv1 specification. (B) Eya, Dimm, and Nplp1 expression in control VNCs at stage AFT, showing that Eya is expressed in all four neurons of the Ap clusters and the dAp cells. Dimm is expressed in two out of four Ap cluster cells and the dAp cells, while Nplp1 is expressed in one neuron (Tv1 cell) of each Ap cluster and the dAp cells. (C) In *lbe* mutants Eya, Dimm and Nplp1 expression is lost in the Ap clusters, whereas their expression in dAp cells is unchanged. (D–E) GFP (*lbe(K)-EGFP*), Dpn, Col, and Nab expression in NB5-6T at St14 showing that Col expression is lost in *lbe* mutants while Nab expression is unaffected. (F, G) GFP, Dpn, Lbe, and Nab expression in NB5-6T at St14 in control and *col* mutants, showing that Lbe expression is not affected in *col* mutants. (H, I) GFP, Dimm, Nplp1, and Eya expression in the Ap cluster at stage AFT in control, reveals loss of Eya, Dimm, and Nplp1 in *lbe* mutants. (J, K) GFP, Lbe, Eya, and Nab expression in the Ap cluster at St16 reveals no effect on Lbe expression in *col* mutants. (L, M) GFP, Lbe, Col, Dpn expression in NB5-6T at St12 and St13 reveals that Lbe is expressed prior to the onset of Col expression. (N, O) Eya, Col, Dimm, and Nplp1 expression at St17 and AFT in the dAp cells of control and *lbe* mutants, revealing no difference in cell fate specification with respect to Nplp1 expression. Genotypes: (B) *OregonR*. (C) *lbe*^*12C005*^/*Df(lbl-lbe)B44*. (D, L, M) *lbe(K)-EGFP*. (E) *lbe(K)-EGFP*/+; *lbe*^*12C005*^/*Df(lbl-lbe)B44*. (F) *lbe(K)-EGFP*/TTG homozygous. (G) *col*^1^/*col*^3^; *lbe(K)-EGFP*/+.

Having identified *grn* as a key spatial regulator upon which the temporal factors act to specify a dAp fate, we attempted to find a counterpart of *grn* in Tv1 fate specification. Recently we performed a large-scale forward genetic screen looking for genes critical for Tv4/FMRFa cell fate which resulted in the identification of additional genes controlling NB5-6T development [31]. One of the mutants identified in this genetic screen, by its loss of *FMRFa-EGFP* expression, was mapped to *ladybird early* (*lbe*) (mammalian *Lbx1/2*). This EMS allele, *lbe*^*12C005*^, has a nonsense mutation at amino acid 29 (a likely null allele) [31], and was placed over deletion *Df(lbl-lbe)B44* to avoid genetic background problems (hereafter referred to as *lbe* mutants). In *lbe* mutants, we observe a complete loss of Eya, Dimm, and Nplp1 (Fig 7A and 7B). Strikingly, we find that *lbe* does not affect dAp neurons (Fig 7A, 7B, 7M and 7N).

In order to further characterize the loss-of-function phenotype of *lbe*, we analysed the expression of other key regulators acting during Ap cluster specification. In *lbe* mutants, we observed normal expression of the sub-temporal factor Nab (Fig 7C and 7D) [14]. However, we observed complete loss of Col, Eya, Dimm, and Nplp1 expression (Fig 7A–7D, 7G and 7H). The loss of Col expression in *lbe* mutants prompted us to reciprocally address Lbe expression in *col* mutants. We observed normal Lbe expression as well as normal Nab expression in *col* mutants (Fig 7E and 7F). As previously described [6], *col* mutants show complete loss of Eya (Fig 7I and 7J). As anticipated, temporal expression analysis revealed that Lbe expression precedes Col expression (Fig 7K and 7L), in line with previous studies showing Lbe expression already at St9 in the NB5-6T [32]. Hence, Lbe is expressed in NB5-6T prior to the onset of any Ap cluster determinants and is critical for the activation of the *col*→*ap*/*eya*→*dimm* terminal selector cascade.

### *lbe* Acts in a Feedforward Manner with *col* in the Tv1 Specification Cascade

**Fig 8:**
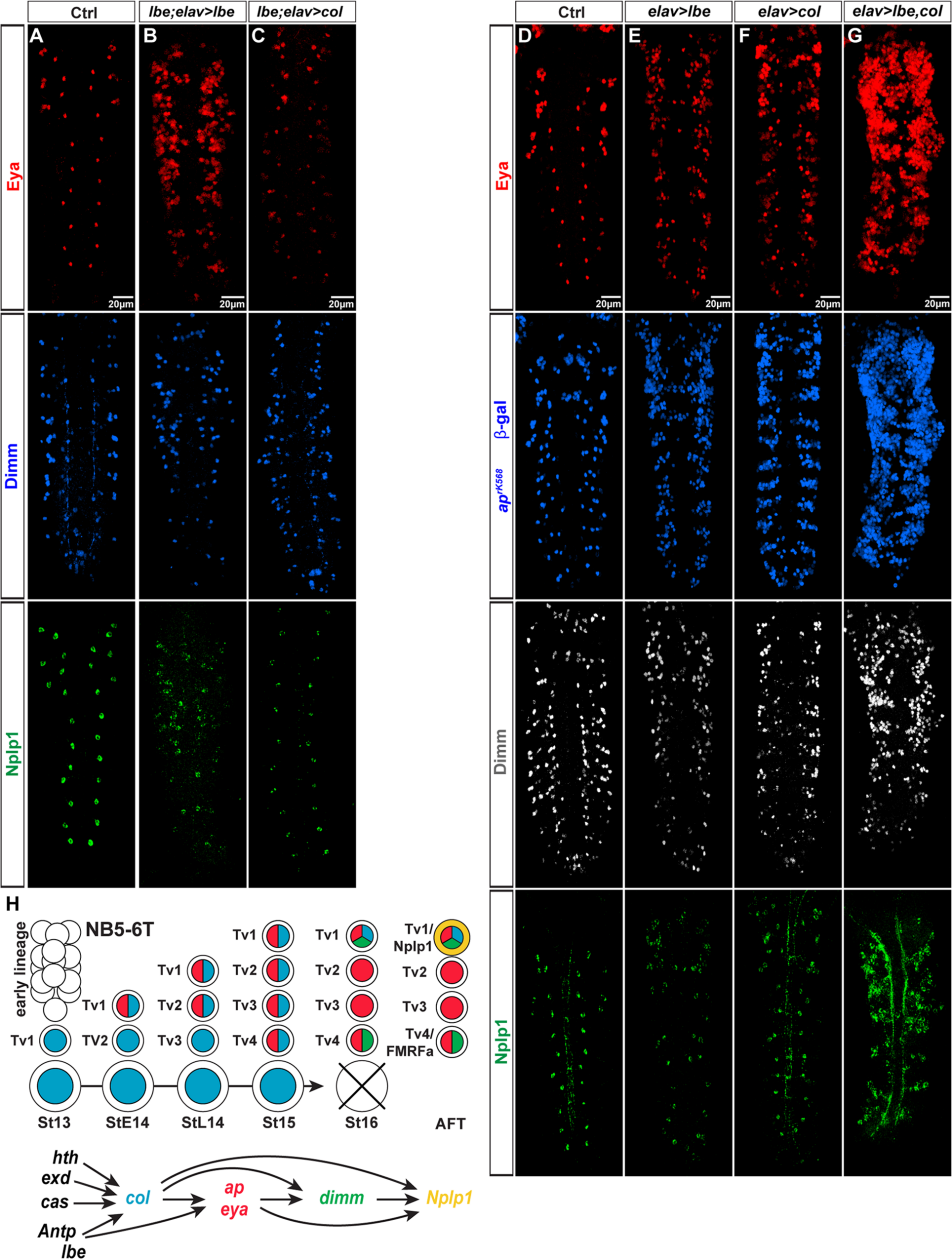
*lbe* is critical for Ap cluster formation and Tv1 specification. (A–C) Expression of Eya, Dimm, and Nplp1 in control and in *lbe* mutants rescued with *UAS-lbe*, or cross-rescued with *UAS-col*, driven from *elav-Gal4*. Rescue of *lbe* mutants by misexpression of *UAS-lbe* can rescue the mutant phenotype in the Ap clusters with respect to Nplp1 expression and gives rise to more Eya positive cells. In contrast, the cross-rescue of *lbe* by misexpression of *UAS-col* fails to rescue the Ap cluster expression of Eya, Dimm, and Nplp1. (D–G) Expression of Eya, βgal (*ap*^*rK568*^), Dimm, and Nplp1. Single misexpression of *lbe* or *col* results in some ectopic Eya, βgal (*ap*^*rK568*^), Dimm, and Nplp1 expression. Co-misexpression of *lbe* and *col* results in a dramatic increase of ectopic Eya, βgal (*ap*^*rK568*^), Dimm, and Nplp1 expression. (H) Model of a potential feed-forward cascade for the Nplp1 specification. *lbe* both activates *col* and potentially feeds forward on downstream targets such as *eya* and *ap*. Genotypes: (A) *OregonR*. (B) *UAS-col*/+; *lbe*^*12C005*^/*Df(lbl-lbe)B44*, *elav-Gal4*. (C) *UAS-lbe*/+; *lbe*^*12C005*^/*Df(lbl-lbe)B44*, *elav-Gal4*. (D) *OregonR*. (E) *ap*^*rK568*^/+; *elav-Gal4*/*UAS-lbe*. (F) *ap*^*rK568*^/+; *elav-Gal4*/*UAS-col*.(G) *UAS-col*/*ap*^*rK568*^; *elav-Gal4*/*UAS-lbe*.

*lbe* regulates *col*, but is this the only role that *lbe* plays, or does it play multiple roles, perhaps acting on targets downstream of *col*? To address this, we attempted to cross-rescue *lbe* using *elav-Gal4* driving *UAS-col*. First, as a control, we rescued *lbe* mutants with *UAS-lbe*, and, as anticipated, this resulted in rescue of thoracic lateral Eya/Dimm/Nplp1 cells (Fig 8A and 8B). Next, we attempted to cross-rescue *lbe* with *UAS-col*, but did not observe any thoracic lateral Eya/Dimm/Nplp1 cells (Fig 8C). These results indicate that *lbe* plays roles in addition to activating *col*, perhaps acting downstream together with *col*. To test this idea, we misexpressed *lbe* and *col* alone, and compared this to the effects of combinatorial misexpression. We noted that each gene alone could trigger ectopic *ap*^*lacZ*^/Eya/Dimm/Nplp1 expression. However, their combinatorial action was striking, with vast numbers of ectopic *ap*^*lacZ*^/Eya cells (Fig 8D–8G). Interestingly, only a subset of ectopic *ap*^*lacZ*^/Eya cells co-expressed Dimm/Nplp1, which may be explained by the fact that *lbe* and *col* are also critical for the Ap cluster Tv2 and Tv3 cell fate: non-neuropeptide expressing interneurons. Finally, we addressed whether *lbe* is regulated by other Tv1 upstream regulators, and stained for Lbe in *cas*, *hth* and *Antp* mutants. This revealed no effects on Lbe expression in any of these three mutants (S1A–S1F Fig). Reciprocally, we tested *lbe* mutants for expression of Cas, Hth, and Antp, but did not observe any effects (S1G–S1J Fig).

These results demonstrate that *lbe* acts in parallel to the four other Ap cluster upstream determinants, and acts in a feedforward manner, first activating *col* and subsequently acting with *col* to activate Ap/Eya/Dimm/Nplp1 (Fig 8H).

## Discussion

**Fig 9:**
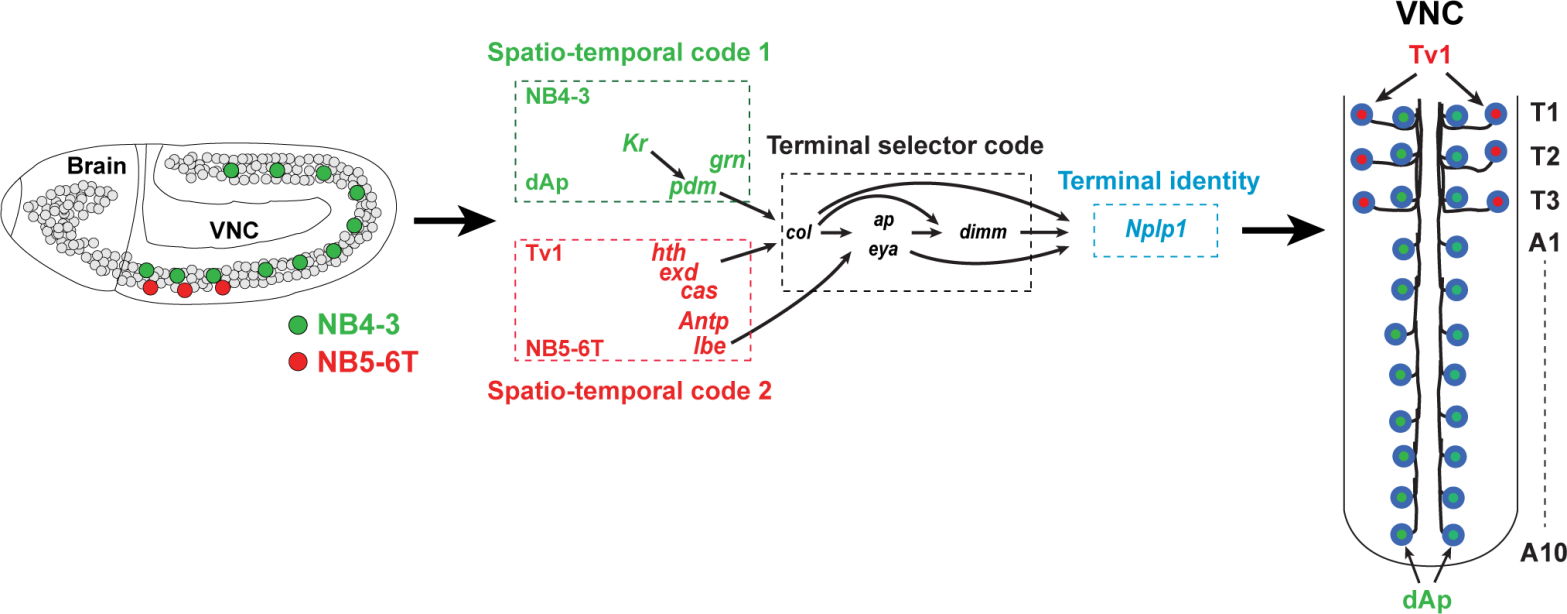
Illustration summarizing the findings. (Left) Lateral view of the early developing *Drosophila* embryonic CNS depicting the thoracic NB5-6T, which generates the Tv1 cells, and the NB4-3, which generates the dAp cells. (Middle-right) Our results reveal that the critical terminal selector gene *col* is activated by different spatio-temporal selector genes acting in the two different NB lineages. In the NB5-6T *col* is activated by the late temporal gene *cas*, together with Hox input, via *Antp*/*hth*/*exd*, and *lbe*. In NB4-3 *col* is activated by the early temporal genes *Kr* and *pdm* and the GATA gene *grn*. Downstream of *col*, the Nplp1 activation cascade in the NB5-6T and NB 4–3 lineages is near identical and acts to specify the related cell fate of the dAp and Tv1 cells. In the NB5-6T lineage, we identified two new players involved in the Nplp1 cell fate specification: *lbe* which activates *col* and feeds forward onto *ap* and *eya*, and *Kr*, which shows a late onset in the Tv1 cell to maintain *col* expression. Hence, different spatiotemporal selector genes acting in cells of a different developmental history triggers a common terminal selector cascade via the key entry point gene *col*.

A number of previous studies have addressed the final steps of neuronal specification with regards to neuropeptide expression in single neuronal lineages in *Drosophila* [6,11,13,14,17,20, 33–36]. However, there are many examples in *Drosophila* of neurons in diverse locations expressing the same neuropeptide [10]. The current study addresses the mechanism by which different upstream cues are integrated to trigger neuropeptide, Nplp1, expression in two spatially and temporally unrelated cells: the Tv1 and dAp cells. We find that the late-born Tv1 cell requires an interplay in the NB5-6T lineage of late temporal selector gene input from *cas* together with spatial input from *Antp*, *lbe*, *hth*, and *exd* to activate *col*; a key trigger gene for the Nplp1 terminal selector cascade (Fig 9). In contrast, the early-born dAp cell requires an input of the early temporal selectors *Kr* and *pdm*, together with the GATA factor *grn*, to activate *col* in NB4-3 (Fig 9). Once *col* is activated in either Tv1 or dAp cell, an identical feed-forward terminal selector cascade plays out downstream of *col* to activate the Nplp1 expression. Hence, the more restricted expression of *grn* and *lbe* acts to refine the broader spatiotemporal cues, triggering a highly restricted terminal selector code, which is initiated by Col expression. Thus, *col* could be viewed as a genetic integrator of different spatiotemporal input.

### Logical Basis for the Different Spatiotemporal Selector Inputs

Thoracic Hox input (Antp), along with the Hox co-factors Exd and Hth, is required for Tv1 specification, while dAp does not require segment-specific Hox input, neither from Antp nor from the posterior bithorax Hox genes [17]. The most obvious reason for this difference is that Tv1 displays a thoracic-specific restriction, while dAp is generated throughout the VNC. Along similar lines, Tv1 cells are born late in NB5-6T and depend upon the late temporal selector Cas, while dAp cells are born early-middle and hence depend upon Kr and Pdm.

In NB5-6T, Col is triggered by a combinatorial code of spatiotemporal selectors (*cas Antp*, *lbe*, *hth*, and *exd*) that to some extent explain its selective expression. However, Col expression is in itself fairly broad and highly dynamic in the developing VNC [6], and hence its expression cannot explain the highly restricted expression of Ap/Eya. However, here *lbe* plays a secondary role, as it acts with *col* to activate Ap/Eya expression. Hence, the highly selective expression of *lbe*, in only a few row 5 NBs, and its feedforward action with *col* combine to refine the action of *col*.

In the case of dAp and NB4-3, we are likely still missing additional upstream and feedforward regulators. First, although Grn contributes to refine the action of Kr/Pdm, the specific activation of expression of Kr, Pdm, and Grn is still not restricted enough to explain the specific triggering of Col in NB4-3. Second, as mentioned above, Col itself is also broadly expressed and needs additional factors to refine its role in NB4-3. Thus, we envision the existence of additional upstream factors in the dAp genetic cascade.

### Expanding Steps of Coherent Feedforward Loops

Expression analysis in mutant and misexpression backgrounds will most often help to place two regulators, X and Y, in relationship to each other. If X expression is not lost in Y mutants, but Y expression is lost in X mutants, one would propose that X regulates Y. However, to address whether or not the only thing X does is to regulate Y, we employ two other approaches: cross-rescue and combinatorial misexpression. The cross-rescue can show, for example, that dAp cells can be fully rescued in *grn* mutants by *col* re-expression, while in contrast, Tv1 cells cannot be rescued in *lbe* mutants by *col* re-expression. Regarding combinatorial misexpression, we observe a striking combinatorial misexpression effect of *lbe*/*col* when compared to either gene alone. Such cross-rescue and co-misexpression experiments prompts us to postulate a direct linear and non-feedforward regulation of *col* by *grn* in dAp cells. In contrast, for Tv1 cells we propose feedforward regulation of *lbe* on *col*, and subsequently with *col* on *ap*/*eya*. Such loops, i.e., *X*→YX→*Z*, are denoted coherent feedforward loops, and are common in *Escherichia coli* and yeast gene regulatory networks [37]. Coherent feedforward loops act as regulatory timing devices and allow for gene X to carry different regulatory output (or meaning) at successive developmental time-points. *col* is a salient example of this; its transient expression in NB5-6T triggers an initial “generic” Ap/Eya interneuron cell fate in the four Ap cluster neurons, while its maintained expression (specifically in Tv1) acts to propagate the terminal selector cascade that ultimately results in the activation of Nplp1 [6,14].

Coherent feedforward loops have also been identified during nervous system development in other animals, including *Caenorhabditis elegans* [38,39]. With regards to neuropeptide cell specification, we now find increasingly longer loops; five steps between Kr and Nplp1 in dAp cells, and ranging in developmental time from St10 to late embryonic stage. The presence of coherent feedforward loops has not been extensively tested in vertebrate systems, primarily because cross-rescue and multiple combinatorial misexpression experiments are technically challenging in these systems. But it is tempting to speculate that coherent feedforward loops are extensively utilized by more complex systems, and that the number of regulatory levels in these loops may increase with evolutionary complexity.

## Materials and Methods

### Fly Stocks

*lbe*^*12C005*^ [31]. *Df(lbl-lbe)B44*, *UAS-lbe*, and *ladybird early* fragment K driving *lacZ* (referred to as *lbe(K)-lacZ*) (provided by C. Jagla) [32]. *lbe(K)-EGFP* [40]. *elav-Gal4* (provided by A. DiAntonio) [41]. *prospero-Gal4* (F. Matsuzaki, Kobe, Japan). *cas*^*Δ1*^ and *cas*^*Δ3*^ (provided by W. Odenwald) [42]. *UAS-nls-myc-EGFP* (referred to as *UAS-nmEGFP*) [11]. *col*^*1*^, *col*^*3*^ [43] and *UAS-col* (provided by A. Vincent) [44]. *hkb*^*5953*^ (referred to as *hkb*^*lacZ*^) [45]. *UAS-ap* and *ap*^*md544*^ (referred to as *ap*^*Gal4*^)[46]. *ap*^*rK568*^ (referred to as *ap*^*lacZ*^) [47]. *UAS-grn-HA* (#F001916; provided by FlyORF). *grh*^*IM*^ [48]. *hb*^*P1*^, *hb*^*FB*^ and *Kr*^*1*^, *Kr*^*CD*^ [27], *unpg*^1912-r37^ *= unpg-lacZ* (provided by C.Q. Doe) [23]. *Antp*^*12*^ (provided by F. Hirth) [49]. *ind-lacZ* and *en-lacZ* (provided by H. Reichert). *grn-lacZ*, *grn*^*7L12*^, *grn*^*SPJ9*^, *UAS-grn* (provided by J. Castelli-Gair Hombría). *col- dAp-GFP* was generated by inserting a genomic fragment from the *col* gene into the vector pEGFP.attB (provided by K. Basler and J. Bischof) and generating transgenes by PhiC31 transgenic integration (BestGene Inc, California, United States).

From Bloomington Drosophila Stock Center: *Antp*^*25*^ (BL#3020). *Df(2L)ED773* (removes both *nub* and *Pdm2*; BL#7416). *mirr-lacZ* (*mirr*^*B1-12*^; BL#30023). *elav*^*C155*^ = *elav-Gal4* (BL#458). *elav-Gal4* (BL#8765). *hth*^*5E04*^ (BL#4221). *Df(3R)Exel6158* (BL#7637; referred to as *hth*^*Df3R*^). Mutants were maintained over *GFP*- or *YFP*-marked balancer chromosomes. As wild type, *w*^*1118*^ or *OregonR* was used. Staging of embryos was performed according to Campos-Ortega and Hartenstein [50].

### Exploratory Screen to Study Nplp1 Specification

The following transcription factor mutants were scored for changes in Nplp1 expression, without any apparent effects: *escargot (esg)*, *shuttle craft (stc)*, *elbow/No ocelli (el/noc)*, *rotund (rn)*, *eagle (eg)*, *kruppel homolog (kr h)*, *knirps (kni)*, *schnurri (shn)*, *klumpfuss (klu)*, *zfh2*, *dachshund (dac)*, *defective proventriculus (dve)*, *seven up (svp)*, *vein (vn)*, *beadex (bx)*, *scribbler (sbb)*.

### Immunohistochemistry

Primary antibodies were: Guinea pig a-Deadpan (1:1,000) (provided by J.B. Skeath). Rabbit a-ß-Gal (1:5,000; ICN-Cappel, Aurora, Ohio, US). Rabbit a-GFP (1:500; Molecular Probes, Eugene, OR, US). Guinea pig a-Col (1:1,000), guinea pig a-Dimm (1:1,000), chicken a-proNplp1 (1:1000), and rabbit a-proFMRFa (1:1,000). Rat a-Grh (1:1,000). Rabbit a-Cas (1:250) (provided by W. Odenwald). Rat mAb a-GsbN (1:10) (provided by R. Holmgren). Mouse a-Nubbin (referred to in the figure as Nub [Pdm]; 1:20) (provided by Steve Cohen). Mouse mAb a-Dac dac2–3 (1:25), mAb a-Antp (1:10), mAb a-Pros MR1A (1:10), mAb a-Eya 10H6 (1:250) (Developmental Studies Hybridoma Bank, Iowa City, Iowa, US). Guinea pig anti-Odd (1:500); guinea pig anti-Runt (both provided by M. Ruiz and D. Kosman). Rat a-Msh (1:500) (provided by Z. Paroush) [51].

### Confocal Imaging and Data Acquisition

Zeiss LSM 700 or Zeiss META 510 Confocal microscopes were used for fluorescent images; confocal stacks were merged using LSM software or Adobe Photoshop. Statistic calculations were performed in Graphpad prism software (v4.03). To address statistical significance, Student's *t* test or nonparametric Mann-Whitney U test or Wilcoxon signed rank test, in the case of non-Gaussian distribution of variables, was used. Images and graphs were compiled in Adobe Illustrator.

## Supporting Information

S1 Data Data underlying Figs 3L, 3N, 4G, 5, 6E, 6F and S5D. (XLSX)

S1 Fig *lbe* acts in parallel with *Antp*, *cas*, and *hth*. (A–F) GFP/βgal, Col, Nab, and Lbe expression in the NB5-6T at St14 in control and *Antp*, *cas*, and *hth* mutants. *Antp* and *hth* mutants show loss of Col expression, while Lbe expression is not affected. (D) *cas* mutants show, in addition to a negative Col expression, a loss of Nab expression, since *cas* regulates *nab* via the sub-temporal gene *sqz*. Lbe expression is however not affected. (G-J) Staining against Antp, Cas, and Hth at St14 in NB5-6 of control and *lbe* mutants shows that neither of these three factors are affected in *lbe* mutants. Genotypes: (A) *lbe(K)-GFP*. (B) *lbe(K)-GFP*/+; *Antp*^*25*^/*Antp*^*12*^. (C) *lbe(K)-GFP*. (D) *lbe(K)-GFP*/+; *cas*^*Δ1*^/*cas*^*Δ3*^. (E) *lbe(K)-lacZ*. (F) *lbe(K)-lacZ*/+; *hth*^*5E04*^/*hth*^*Df*^. (G, I) *lbe(K)-GFP*. (H, J) *lbe(K)-GFP*/+; *lbe*^*12C005*^/*Df(lbl-lbe)B44*.
(TIF)

S2 Fig Origin of extra dAp cells in *hb* mutant background. Co-staining for GFP and Eya of the *col-dAp-GFP* enhancer (to visualize the NB4-3 from which dAp cell originated) in (A) control and (B) hb mutant background. Both the bonafide dAp and the supernumerary one are GFP positive. Thus, the supernumerary dAp generated in *hb* mutant originate from the NB4-3. Genotypes: *col-dAp-GFP/+; hb*^*P1*^, *hb*^*FB*^.
(TIF)

S3 Fig Castor is not expressed in the NB4-3 early lineage and dAp is generated in a type I division mode. (A and B) Co-staining for GFP and Cas of the *col-dAp-GFP* enhancer (to visualize the NB4-3 lineage from which dAp cell originated) at Stage 12 and 13 to analyze the expression of Cas in the NB4-3 lineage when it is generating the dAp neuron. Cas is not expressed in the NB4-3 early lineage (C) Co-staining for GFP, Dimm, and Eya of the *col-dAp-GFP* enhancer (to visualize the NB4-3 lineage from which dAp cell originated) in *spodo* mutant background. Additional dAp cell express GFP, Eya, and Dimmed in Spodo mutant. Genotypes: (A) *col-dAp-GFP*; *col-dAp-GFP*. (B) *col-dAp-GFP/+*; *spdo*^*6104*^*/spodo*^*6104*^.
(TIF)

S4 Fig Grn expression in the NB5-6 lineage at two different stages. (A) Co-staining for βgal of the *grn-lacZ* and Eya to analyze the expression of *grn-lacZ* at the Ap cluster at St16. We do not find *grn-lacZ* expression in the NB5-6 lineage at St 16. (B) Co-staining for GFP of the *lbe(K)-GFP* (to visualize the NB5-6 lineage) together with βgal for the *grn-lacZ* construct at Stage 15 to analyze the expression of *grn-lacZ* in the NB5-6 lineage. We do not find *grn-lacZ* expression in the NB5-6 lineage at St 15. Genotypes: (A) *grn-lacZ/+*. (B) *lbe(K)-GFP/ grn-lacZ*.
(TIF)

S5 Fig Overexpression of *grn*. (A-C) Co-staining for Eya, Nplp1 and Col in (A) control, (B) *pros-Gal4>UAS-grn*, and (C) *elav-Gal4>UAS-grn* genetic background. Overexpression of *grn* is not able to induce ectopic dAp neurons. (D) Quantification of Nplp1 and Eya expressing dAp cells in control, *prospero-Gal4>UAS-grn*, and *elav-Gal4>UAS-grn* genetic background VNCs (n = 7 VNCs for *pros>grn* for Eya cell quantification; for all others, n >10 VNCs; asterisks denote *p* < 0.05, Student´s two-tailed *t*-test; see S1 Data) Genotypes: (A) *OregonR*. (B). *prospero-Gal4/UAS-grn*. (C) *elav-Gal4/UAS-col*.
(TIF)

S6 Fig Origin of supernumerary Eya cells in *col* and *lbe* co-misexpression. (A, B) Co-staining for βgal, Dimm, Eya, and GsbN of the *mirror-lacZ* construct in (A) control and (B) *elav-Gal4>UAS-col*, *UAS-lbe* genetic background. White dotted lines represent the Gsbn compartment whereas magenta dotted lines represent the Mirr compartment. Supernumerary Eya cells generated by *UAS-col*, *UAS-lbe* co-misexpression originate from lineages generated by NBs in row 5 (Gsbn) as well from lineages generated by NBs in row 1, 2 and 3 (*mirr-lacZ*). Genotypes: (A) *mirr-lacZ/ UAS-col; UAS-lbe*. (B) *elav-Gal4;; mirr-lacZ/ UAS-col*, *UAS-lbe*.
(TIF)

S7 Fig *Kr*, *pdm*, and *grn* are not required for dAp cell survival. (A–C) Co-staining for Dimm and Eya in *Kr*, *pdm*, and *grn* mutants, in which cell death has been impaired by *Df(3R)H99* or by expression of cell death blocker *UAS-p35*. dAp cells are lost in mutants and not restored by cell death impairment.
(TIF)

## Abbreviations

AFT: air-filled trachea
Ap: Apterous
Cas: Castor
CNS: central nervous system
Eya: Eyes absent
GMC: ganglion mother cell
Grn: Grain
NB: neuroblast
VNC: ventral nerve cord
