# Supplementary figures and images for: Neuronal Cell Fate Specification by the Convergence of Different Spatiotemporal Cues on a Common Terminal Selector Cascade
Source: PLoS Biol. 2016 May 5;14(5):e1002450. doi: 10.1371/journal.pbio.1002450 (PMC4858240; doi:10.1371/journal.pbio.1002450)

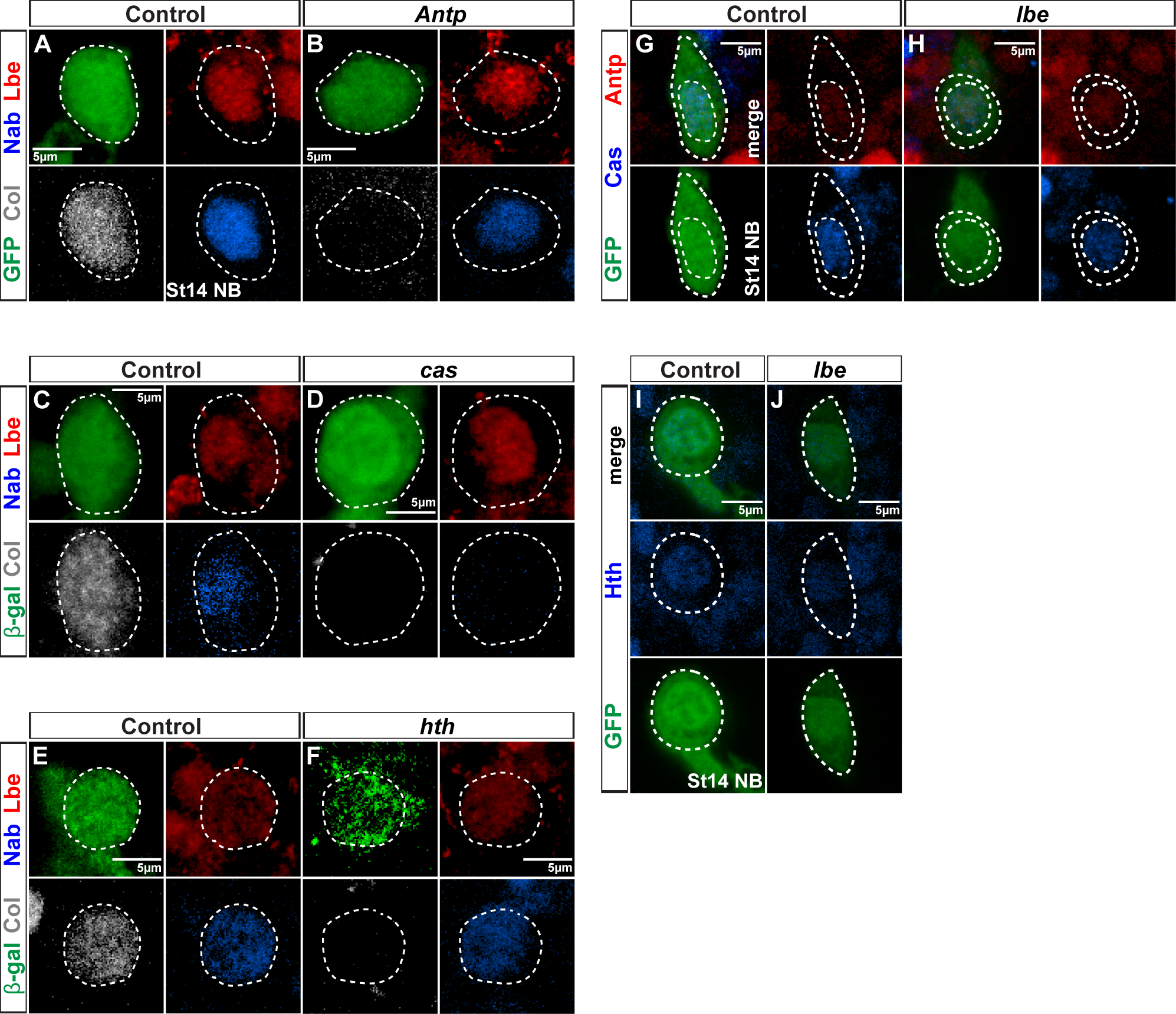

Supplement: S1 Fig — (A–F) GFP/βgal, Col, Nab, and Lbe expression in the NB5-6T at St14 in control and Antp, cas, and hth mutants. Antp and hth mutants show loss of Col expression, while Lbe expression is not affected. (D) cas mutants show, in addition to a negative Col expression, a loss of Nab expression, since cas regulates nab via the sub-temporal gene sqz. Lbe expression is however not affected. (G-J) Staining against Antp, Cas, and Hth at St14 in NB5-6 of control and lbe mutants shows that neither of these three factors are affected in lbe mutants. Genotypes: (A) lbe(K)-GFP. (B) lbe(K)-GFP/+; Antp25/Antp12. (C) lbe(K)-GFP. (D) lbe(K)-GFP/+; casΔ1/casΔ3. (E) lbe(K)-lacZ. (F) lbe(K)-lacZ/+; hth5E04/hthDf. (G, I) lbe(K)-GFP. (H, J) lbe(K)-GFP/+; lbe12C005/Df(lbl-lbe)B44. (TIF) [file pbio.1002450.s002.tif]

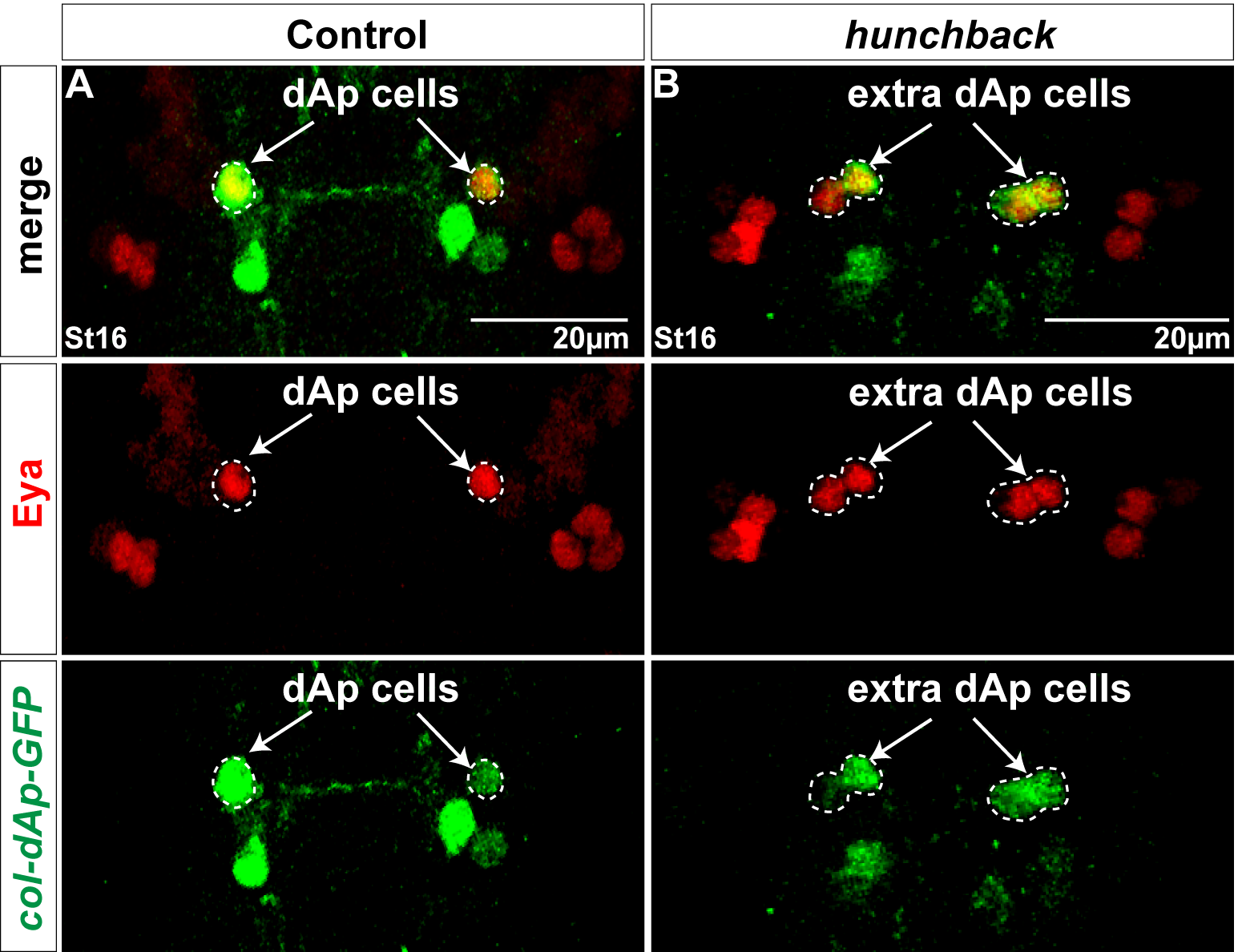

Supplement: S2 Fig — Co-staining for GFP and Eya of the col-dAp-GFP enhancer (to visualize the NB4-3 from which dAp cell originated) in (A) control and (B) hb mutant background. Both the bonafide dAp and the supernumerary one are GFP positive. Thus, the supernumerary dAp generated in hb mutant originate from the NB4-3. Genotypes: col-dAp-GFP/+; hbP1, hbFB. (TIF) [file pbio.1002450.s003.tif]

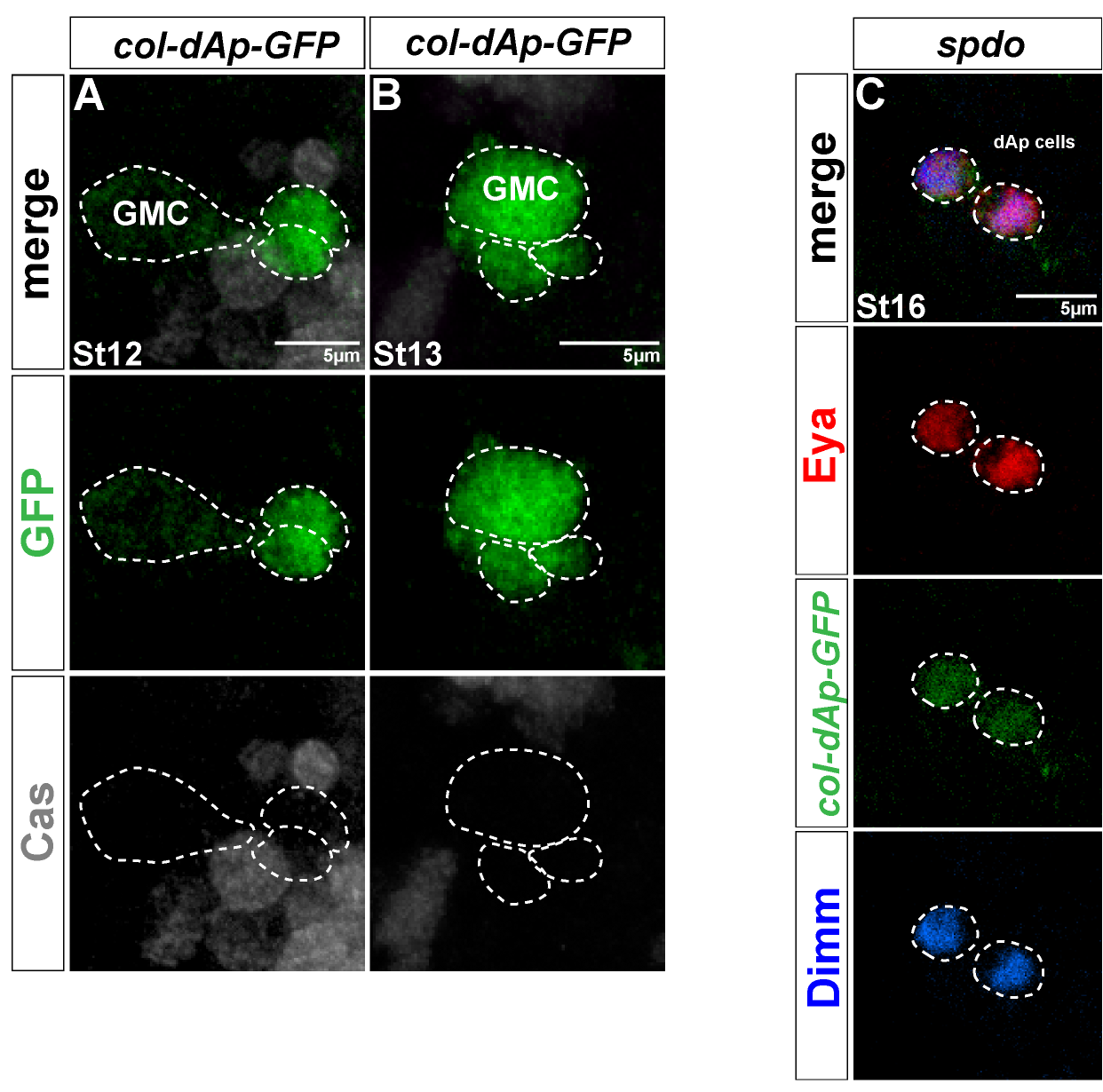

Supplement: S3 Fig — (A and B) Co-staining for GFP and Cas of the col-dAp-GFP enhancer (to visualize the NB4-3 lineage from which dAp cell originated) at Stage 12 and 13 to analyze the expression of Cas in the NB4-3 lineage when it is generating the dAp neuron. Cas is not expressed in the NB4-3 early lineage (C) Co-staining for GFP, Dimm, and Eya of the col-dAp-GFP enhancer (to visualize the NB4-3 lineage from which dAp cell originated) in spodo mutant background. Additional dAp cell express GFP, Eya, and Dimmed in Spodo mutant. Genotypes: (A) col-dAp-GFP; col-dAp-GFP. (B) col-dAp-GFP/+; spdo6104/spodo6104. (TIF) [file pbio.1002450.s004.tif]

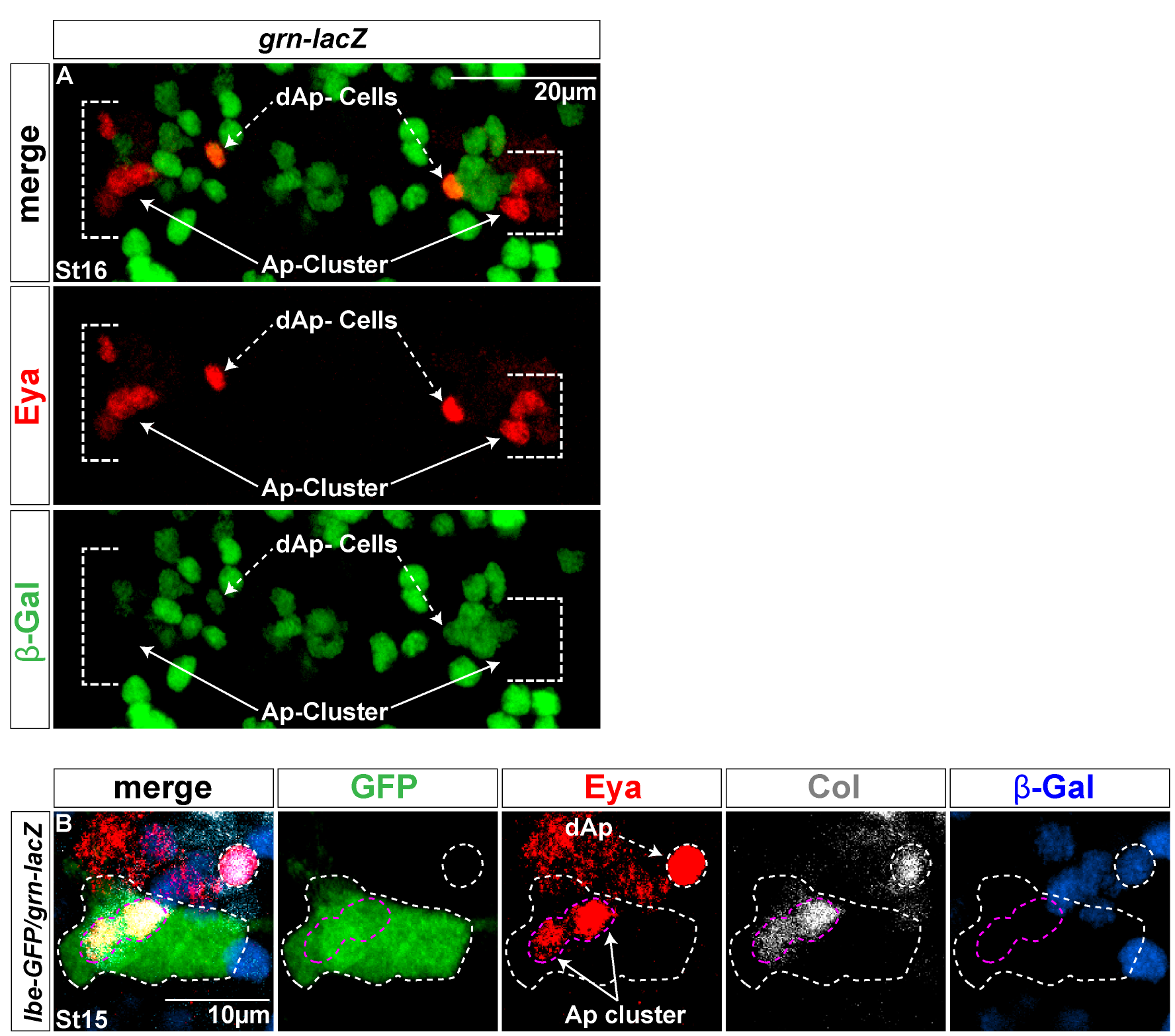

Supplement: S4 Fig — (A) Co-staining for βgal of the grn-lacZ and Eya to analyze the expression of grn-lacZ at the Ap cluster at St16. We do not find grn-lacZ expression in the NB5-6 lineage at St 16. (B) Co-staining for GFP of the lbe(K)-GFP (to visualize the NB5-6 lineage) together with βgal for the grn-lacZ construct at Stage 15 to analyze the expression of grn-lacZ in the NB5-6 lineage. We do not find grn-lacZ expression in the NB5-6 lineage at St 15. Genotypes: (A) grn-lacZ/+. (B) lbe(K)-GFP/ grn-lacZ. (TIF) [file pbio.1002450.s005.tif]

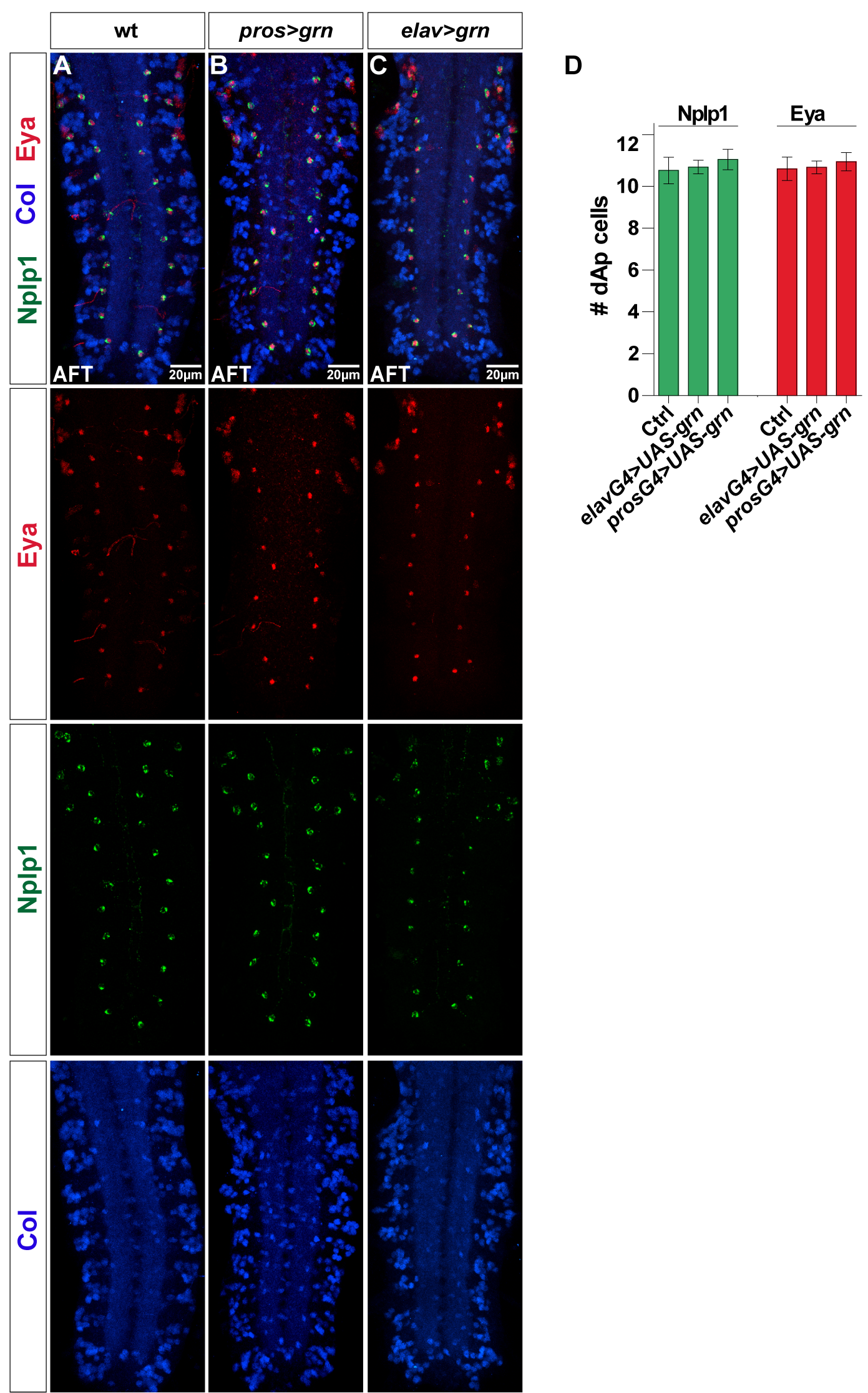

Supplement: S5 Fig — (A-C) Co-staining for Eya, Nplp1 and Col in (A) control, (B) pros-Gal4>UAS-grn, and (C) elav-Gal4>UAS-grn genetic background. Overexpression of grn is not able to induce ectopic dAp neurons. (D) Quantification of Nplp1 and Eya expressing dAp cells in control, prospero-Gal4>UAS-grn, and elav-Gal4>UAS-grn genetic background VNCs (n = 7 VNCs for pros>grn for Eya cell quantification; for all others, n >10 VNCs; asterisks denote p < 0.05, Student´s two-tailed t-test; see S1 Data) Genotypes: (A) OregonR. (B). prospero-Gal4/UAS-grn. (C) elav-Gal4/UAS-col. (TIF) [file pbio.1002450.s006.tif]

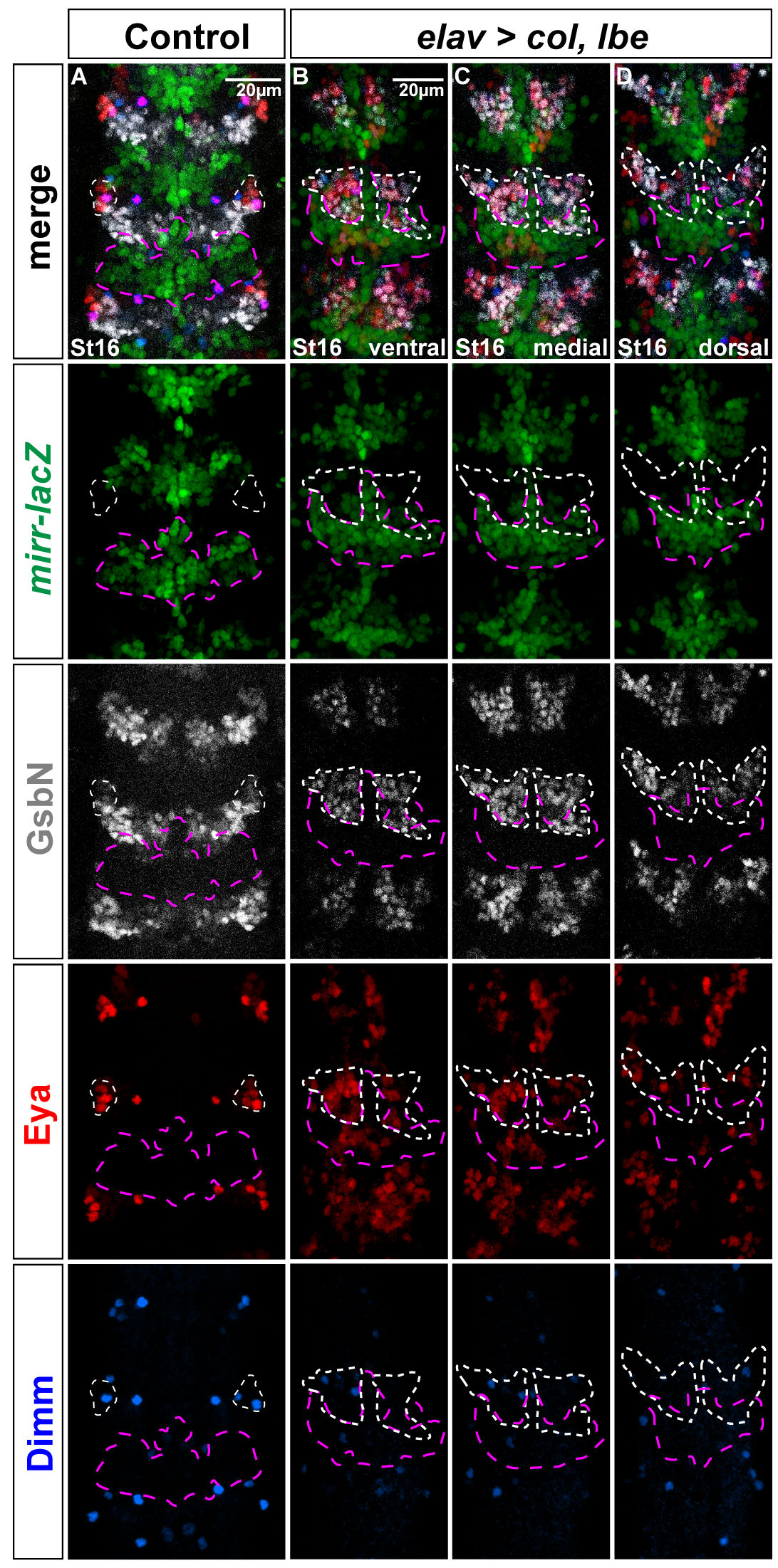

Supplement: S6 Fig — (A, B) Co-staining for βgal, Dimm, Eya, and GsbN of the mirror-lacZ construct in (A) control and (B) elav-Gal4>UAS-col, UAS-lbe genetic background. White dotted lines represent the Gsbn compartment whereas magenta dotted lines represent the Mirr compartment. Supernumerary Eya cells generated by UAS-col, UAS-lbe co-misexpression originate from lineages generated by NBs in row 5 (Gsbn) as well from lineages generated by NBs in row 1, 2 and 3 (mirr-lacZ). Genotypes: (A) mirr-lacZ/ UAS-col; UAS-lbe. (B) elav-Gal4;; mirr-lacZ/ UAS-col, UAS-lbe. (TIF) [file pbio.1002450.s007.tif]

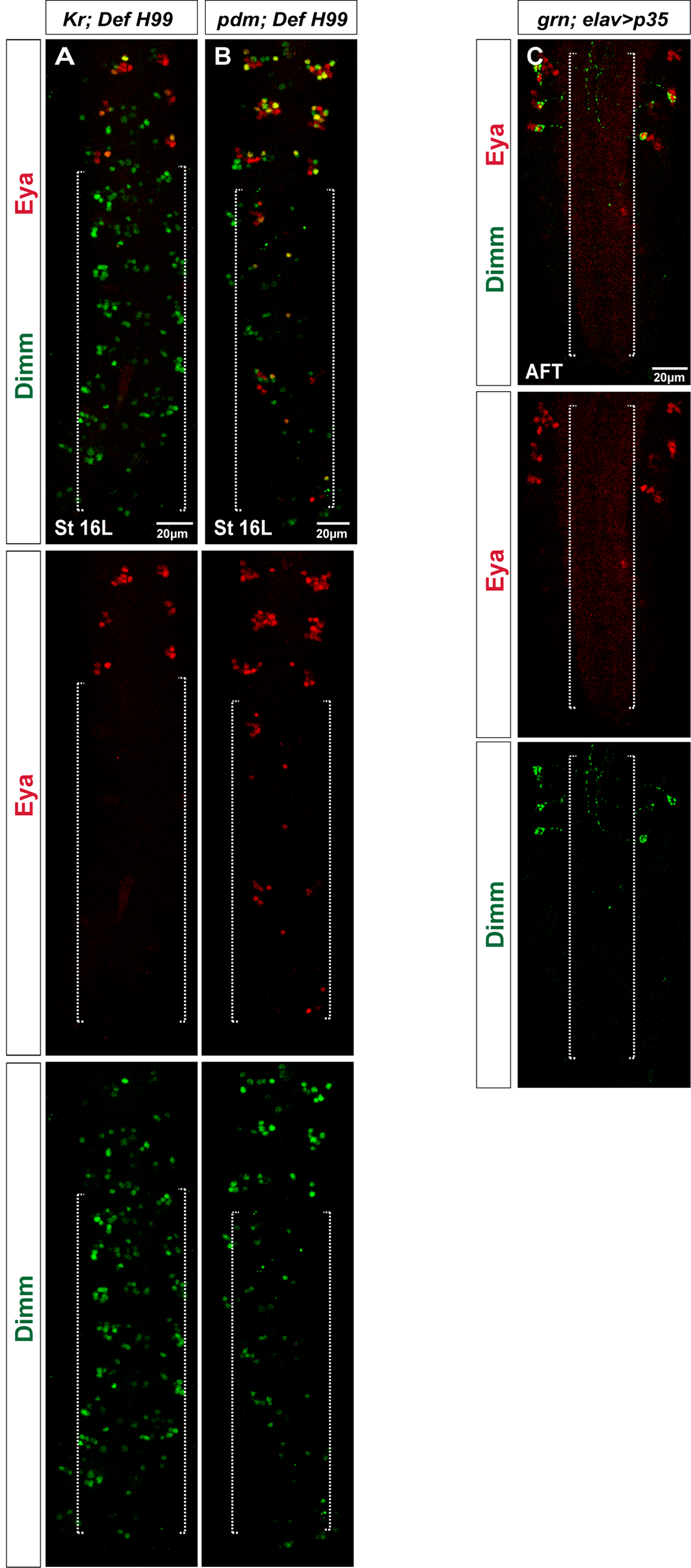

Supplement: S7 Fig — (A–C) Co-staining for Dimm and Eya in Kr, pdm, and grn mutants, in which cell death has been impaired by Df(3R)H99 or by expression of cell death blocker UAS-p35. dAp cells are lost in mutants and not restored by cell death impairment. (TIF) [file pbio.1002450.s008.tif]
